# PCR Biases Distort Bacterial and Archaeal Community Structure in Pyrosequencing Datasets

**DOI:** 10.1371/journal.pone.0043093

**Published:** 2012-08-15

**Authors:** Ameet J. Pinto, Lutgarde Raskin

**Affiliations:** Department of Civil and Environmental Engineering, University of Michigan, Ann Arbor, Michigan, United States of America; Uppsala University, Sweden

## Abstract

As 16S rRNA gene targeted massively parallel sequencing has become a common tool for microbial diversity investigations, numerous advances have been made to minimize the influence of sequencing and chimeric PCR artifacts through rigorous quality control measures. However, there has been little effort towards understanding the effect of multi-template PCR biases on microbial community structure. In this study, we used three bacterial and three archaeal mock communities consisting of, respectively, 33 bacterial and 24 archaeal 16S rRNA gene sequences combined in different proportions to compare the influences of (1) sequencing depth, (2) sequencing artifacts (sequencing errors and chimeric PCR artifacts), and (3) biases in multi-template PCR, towards the interpretation of community structure in pyrosequencing datasets. We also assessed the influence of each of these three variables on α- and β-diversity metrics that rely on the number of OTUs alone (richness) and those that include both membership and the relative abundance of detected OTUs (diversity). As part of this study, we redesigned bacterial and archaeal primer sets that target the V3–V5 region of the 16S rRNA gene, along with multiplexing barcodes, to permit simultaneous sequencing of PCR products from the two domains. We conclude that the benefits of deeper sequencing efforts extend beyond greater OTU detection and result in higher precision in β-diversity analyses by reducing the variability between replicate libraries, despite the presence of more sequencing artifacts. Additionally, spurious OTUs resulting from sequencing errors have a significant impact on richness or shared-richness based α- and β-diversity metrics, whereas metrics that utilize community structure (including both richness and relative abundance of OTUs) are minimally affected by spurious OTUs. However, the greatest obstacle towards accurately evaluating community structure are the errors in estimated mean relative abundance of each detected OTU due to biases associated with multi-template PCR reactions.

## Introduction

Next-generation sequencing (NGS) technologies increasingly are being applied in microbial diversity studies by either targeting the 16S rRNA gene [1] or by direct sequencing of genomic DNA or RNA (through cDNA sequencing) extracted from environmental samples [2]. In particular, the use of 16S rRNA gene based amplicon sequencing has become common in studies of microbial communities in natural [3]–[5] and engineered [6], [7] ecosystems. Several massively parallel sequencing options are currently available [2], but thus far only Roche’s 454 [8] and IIumina’s GAIIx, HiSeq, and MiSeq 9–11 platforms have been utilized for 16S rRNA gene based amplicon sequencing. Two key features that make these sequencing technologies very attractive include (1) deep sequencing to explore the diversity that so far has been undetected due to methodological constraints [8], and (2) the ability to multiplex a large number of samples in the same sequencing run through the inclusion of multiplexing barcodes ligated to template specific PCR primers [12]. The benefits of massively parallel sequencing have been accompanied by several methodological challenges. First, the current technologies cannot sequence the entire length of the 16S rRNA gene. Though improvements towards increasing the sequencing length have been rapid, the quality scores deteriorate after the first 250 sequenced nucleotides for the 454-titanium platform [13] and Illumina sequencing currently provides information only up to 200 nucleotides [10]. Therefore, sequencing studies have focused on those hypervariable regions of the 16S rRNA gene from which substantial taxonomic information can be inferred [14], [15] and that allow for discrimination similar to that provided by full length gene sequence analysis [16].

Another focus of the amplicon based sequencing studies has been to eliminate the effects of insertion/deletion type sequencing errors and chimeric PCR artifacts on the estimation of diversity. Approaches to minimize sequencing errors include the use of high quality score thresholds to remove poor quality sequences [13], [17], sequence correction through correction of flowgrams [18], denoising [19], and use of sequence pre-clustering [20]. The elimination of chimeras has largely focused on their detection and removal from large databases of short reads either by comparison to reference datasets of good quality sequences or by comparing each read to the others within each sample library [21]–[23]. Despite these rapid advances, some sequencing errors and chimeras are retained in the processed datasets and severely inflate the estimated richness of the sample [24]. In addition to sequencing errors and chimeras, PCR based methods introduce biases that can affect the results of microbial community structure analyses. For example, a large portion of the microbial diversity in a sampled community may not be captured due to primer mismatches [25]. Additionally, differential amplification efficiencies of the 16S rRNA genes in multi-template PCR reactions [26], [27] can influence the representation of the sampled community by altering relative abundances of detected operational taxonomic units (OTUs) and thus distort the original rank abundance distribution. Such PCR biases can be particularly significant since the current procedure for amplicon-sequencing involves at least two different amplification steps, i.e., PCR amplification during initial sample preparation using template specific primers followed by emulsion PCR (emPCR) on Roche’s 454 or bridge PCR on Illumina platforms, prior to sequencing. In some studies, an additional PCR step has been included to pre-amplify the 16S rRNA gene prior to nested amplification with 454-compatible primers [28], [29]. The amplification efficiency in multi-template PCR reactions is affected by several parameters, such as primer choice [30], the GC content of the target region [31], [32], thermocycling conditions [33], DNA template concentration [26], and the relative abundance of the target sequences. This topic of differential amplification and its effect on the interpretation of community structure based on pyrosequencing data have not been systematically studied thus far.

In this study, we evaluated how errors in mean relative abundances of OTUs resulting from multi-template PCR bias affected the representation of six mock communities constructed by combining 33 bacterial and 24 archaeal 16S rRNA gene sequences in three different ways, each. Additionally, we compared distortions in community structure due to multi-template PCR bias to the biases originating from the presence of spurious OTUs, containing sequences with insertion/deletion type errors and chimeric sequences, some of which are retained in pyrosequencing data despite the use of quality control measures. This study was conducted using data from two independent sequencing runs at two different sequencing depths (as determined by the final number of reads in each sequencing library). In doing so, we also (1) determined the effect of sequencing depth on the taxa detection frequency (defined below), the mean relative abundance of OTUs, and the rank abundance distribution of communities; (2) evaluated the influence of sequencing depth, spurious OTUs, and errors in mean relative abundance on α- and β-diversity metrics; and (3) determined diversity metrics that are more reliable when used in conjunction with pyrosequencing data. The mock communities were designed to include sequences of strains covering broad phylogenetic diversity and variable GC content to evaluate the effects of differences in amplification efficiency, and were tested with newly designed bacterial and archaeal primer sets that target the V3–V5 hypervariable region of the 16S rRNA gene.

## Results and Discussion

### Design and Coverage of Bacterial and Archaeal 16S rRNA Targeted Primers

We modified previously developed primers, Bact-338F/Bact-909R [34], [35] and Arch-340F/Arch-915R [36], [37], targeting the V3–V5 hypervariable region of the 16S rRNA gene for both bacteria and archaea to improve coverage of existing sequences in databases (Table 1 and Figure S1). The choice of the V3–V5 hypervariable region was motivated by previous successful classification of bacterial amplicons in this region [14], [35] and the high correlation between phylogenetic information derived from V3–V5/V4 regions with that from the full length 16S rRNA gene [16]. Based on *in-silico* database searches, the new bacterial primers match approximately 96% of the sequences present in the Ribosomal Database Project (RDP) database (release 10) [55], with greater than 90% coverage of most major phyla (Figure S1). Additionally, the archaeal primers, which were modifications of previous primers [28], [37], [38], matched approximately 87% of the sequences in the RDP database, with high and equivalent coverage of the major archaeal phyla, i.e., *Crenarchaeota* (88%), *Euryarchaeota* (92%), *and Korarchaeota* (90%). We also designed the reverse primers integrated with multiplexing barcodes to minimize the interference of secondary structures, such as hairpins, homo-dimers, and hetero-dimers during PCR reactions, and allow for subsequent sorting of sequences from both archaeal and bacterial libraries into their respective samples (Table S1). The successful use of the new primers and multiplexing barcodes was demonstrated by (1) the effective amplification of the 16S rRNA genes from tested samples, and (2) the recovery of bacterial and archaeal sequences in expected proportions for both sequencing runs. Specifically, the ratios of bacteria:archaea obtained in the two sequencing runs were 63∶37 and 65∶35, similar to the 60∶40 ratio at which the bacterial and archaeal amplicon pools were combined prior to emPCR and sequencing.

To further check the coverage of the newly designed primers, we tested them on DNA extracts from multiple environmental samples. DNA extracted from samples collected from a drinking water distribution system (DWDS), an anaerobic bioreactor (ANBR), mouse gut cecal tissue (MGCT), surface water (SW), a deep sea sample from the Gulf of California (GC), and the Obsidian Pool in Yellowstone National Park (OP-YNP) were tested with the bacterial primers designed in this study. The GC, OP-YNP, and ANBR DNA extracts, as well as DNA extracted from a freshwater aquaculture system (FAS) sample were tested with the archaeal primers. Tables S2 and S3 provide the taxonomic classification of sequences detected in each sample. Despite high coverage of the archaeal primers for the *Korarchaeota* phylum (Figure S1), none of the sequences detected in the OP-YNP sample were classified to this phylum. To evaluate if *Korarchaeota* were not detected due to the primers designed in this study, we tested the OP-YNP DNA extract with published korarchaeal primers in conjunction with general archaeal primers in the following combination: Kora-228F/Univ-1406R, Kora-228F/Kora-1236R, Arch-4F/Kora-1236R, Arch-112F/Kora-1236R [36], [39]. None of these primer combinations yielded *Korarchaeota* sequences. Hence, we conclude that the non-detection of *Korarchaeota* sequences using the archaeal primers was due to the absence of this phylum in the OP-YNP DNA extract. One of the sequences detected in the OP-YNP sample was classified as *Nanoarchaeota* even though the primers showed no perfect matches to any of the *Nanoarchaeota* sequences in the RDP database. Additionally, the archaeal primers were able to capture sequences representing various families within the *Crenarchaeota* phylum in the OP-YNP sample and *Thaumarchaeota* sequences classified within the *Nitrosopumilaceae* family in the GC samples. The primers also captured multiple methanogenic *Euryarchaeota* sequences in the ANBR samples, which were not found in the other three samples tested. Likewise, the bacterial primers were able to detect sequences representing a diverse array of phyla and families in the six environmental samples tested. For example, they were able to detect sequences in the DWDS sample that classified within the *Chlamydiae* phylum. This is noteworthy because some *Chlamydiae* are endosymbionts of amoebae [40], which can harbor and protect bacterial pathogens [41]. The primers also detected many sequences that classified within two different families of the *Aquificae* phylum, which would have been missed with previously used V3–V5 primers [34].

**Table 1:** Bacterial and archaeal primers targeting the V3–V5 region of the sequences used in this study and their respective coverage for the sequences in the RDP database (release 10).

Domain	Primer name	Sequence	Combined coverage of RDP database	Reference
Bacteria	Bact-338F1	CCTACGGG**R**GGCAGCAG	96.4%	This study
	Bact-338F2	AC**WY**CTACGG**RW**GGCTGC		This study
	Bact-338F3	CACCTACGGGTGGCAGC		54
	Bact-909R	CCGTCAATT**YH**TTT**R**AGT		This study
Archaea	Arch-340F	CCCTA**H**GGGG**Y**GCA**S**CA	86.5%	This study
	Arch-915R	G**W**GC**Y**CCCCCG**Y**CAATTC		This study

Degeneracy code: R = A/G, Y = C/T, W = A/T, H = A/C/T.

Phylum and order level coverage for bacteria and archaea are provided in Figure S1.

### The GC Content of a Sample Library Affects the Number of Final Reads

**Figure 1:**
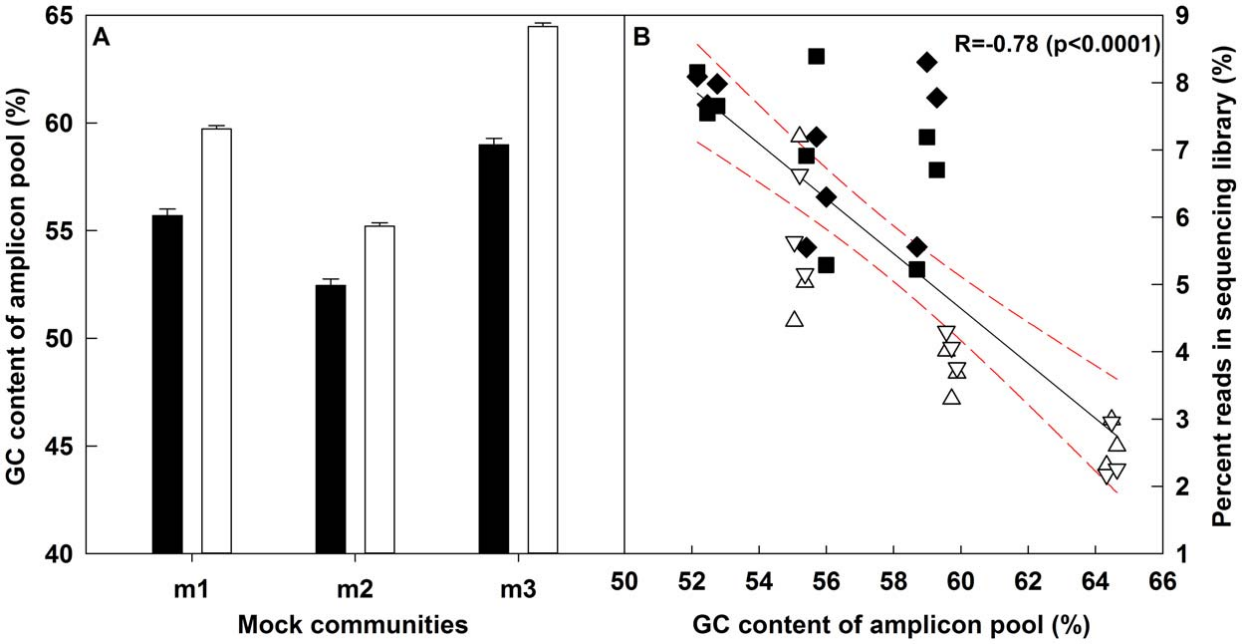
The mean percent GC content of the three bacterial and three archaeal mock communities (A) and the resulting reads attributed to mock community replicates in the final 454-sequencing output expressed as percent reads in sequencing library versus the GC content of the amplicon pool (B). Error bars in panel A represent variation in GC content between replicates of each community resulting from differences in GC content of barcoded reverse primer. Black bars: bacteria, white bars: archaea. The red dotted line in panel B shows the 95% confidence band for the regression line. Black symbols: bacteria, white symbols: archaea. Diamonds (◊) and upper triangle (Δ): large library, Squares (□) and lower triangle (∇): small library.

Previous work has shown that GC content may have a strong effect on PCR [32], [42], [43] and whole genome amplification [44], and on whole genome sequencing using the Illumina [45] and 454-GS-FLX and Titanium platforms [46]. So far, such a bias has not been presented in the literature for amplicon based 454-sequencing. Therefore, we evaluated whether the presence of GC bias affects (1) how a sequence is represented within a sample (i.e., intra-sample GC bias) and/or (2) how a sample is represented within a sequencing library (i.e., inter-sample GC bias). To evaluate the effect of GC content, we exploited the variations in GC contents of the V3–V5 regions of the 33 bacterial and 24 archaeal 16S rRNA gene sequences that were used to construct two sets of three mock communities (Table S4 and Figures S2). Specifically, the mock communities were constructed by dividing the bacterial and archaeal sequences into low, medium, and high GC clusters with 11 bacterial and eight archaeal sequences in each cluster (Figure S2). Sequences within each GC cluster were mixed at three different abundance levels to construct three bacterial and three archaeal mock communities (Figure S2). This resulted in six different mock communities with six different GC contents, three each for bacteria and archaea. We PCR amplified the three replicates of each mock community with reverse primers with three different GC contents resulting in slightly different overall GC contents for each mock community (Figure 1A). Since the PCR products from each mock community replicate were mixed in equimolar proportion prior to emPCR and sequencing, each sample library should have contained an equal number of sequences if GC content of the sample library did not result in any biases. However, we were able to detect a significant correlation between the number of reads in each sample and the overall GC content of the sample (Figure 1B) (*R = 0.78, p<0.0001*) when all bacterial and archaeal sample libraries were considered. The correlation was even stronger when only the archaeal libraries were considered (*R = 0.89, p<0.0001*). This clearly demonstrates that samples with high GC content may be under-represented in a 454-amplicon sequencing output, which is consistent with previous observations for whole genome sequencing [44]. This observation is particularly important since equal sequencing depth, i.e., the number of reads per sample in a multiplexing run, is critical for consistent comparisons of multiple samples. We further evaluated whether any GC content bias could be attributed towards errors in relative abundance of all the detected OTUs (intra-sample bias), but were unable to find any suggestive correlations. Nonetheless, the observations of GC bias at the sample level (inter-sample bias) merit further systematic investigations to determine to what extent GC content can explain errors in mean relative abundance of an OTU (intra-sample bias).

### Effect of Sequencing Depth and Relative Abundance on Taxa Detection Frequency

**Figure 2:**
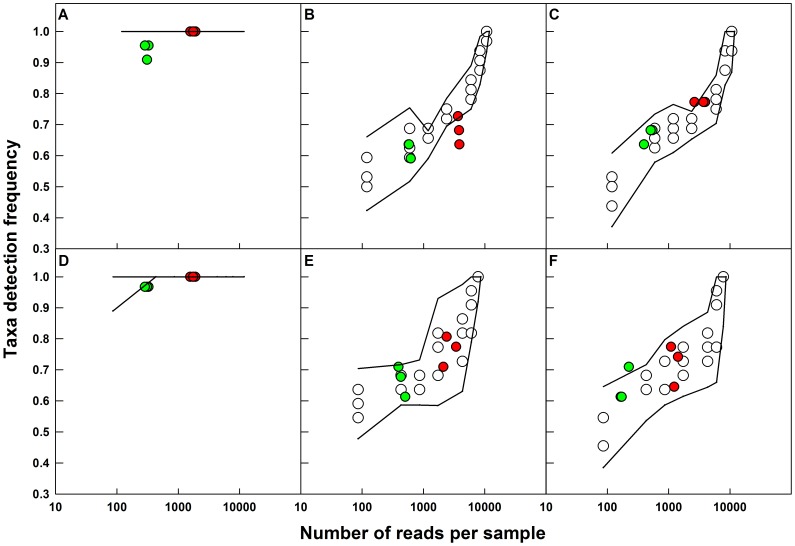
The taxa detection frequency for each of the replicate mock communities at different sequencing depths are compared to detection frequency at different theoretical sampling depths. Open circles: sub-samples of *in-silico* mock communities with varying number of sequences, red circles: large library, green circles: small library, solid lines: 95% confidence interval band for the *in-silico* sub-sampling efforts. A–C: bacteria, D–F: archaea, A/D: mock1, B/E: mock 2, C/F: mock 3. Theoretical taxa detection frequencies for mock community 1 (bacteria and archaea) are 1.0 for most *in-silico* sub-sampling efforts and hence are not shown in panels A and D.

Several β-diversity metrics, e.g., Jaccard, Hamming, unweighted UniFrac [47], use a presence/absence approach, i.e., the level of similarity between two samples is assessed by whether an OTU detected in one sample was found in the other or not. Under such scenarios, the taxa detection frequency (i.e., the ratio of the number of taxa detected over the true number of taxa present in a sample) becomes critical. Many factors may affect the detection frequency. However, since plasmid inserted sequences were used in this study, we were unable to assess the influence of DNA extraction [48] and the effect of the whole genome on PCR amplification of the 16S rRNA gene fragment [49]. Rather, we evaluated how the taxa detection frequency was affected by different OTU abundance distributions by making the probability of detection equal for all sequences in mock community m1 and variable in mock communities m2 and m3, i.e., corresponding to the relative abundance of each sequence in these mock communities (Figure S2). Additionally, we determined the effect of sequencing depth on the taxa detection frequency by independently sequencing the replicate mock communities twice, with the second run providing 6.4±0.8 and 6.1±0.7 fold more reads than the first run for the bacterial and archaeal mock communities, respectively (Table S5). We compared the experimentally observed taxa detection frequencies to the theoretical estimates as determined by random sub-sampling of the *in-silico* mock communities at multiple depths (Figures 2A–2F). The evaluation of taxa detection frequency presented in this section is limited to good sequences, i.e., those sequences that clustered with the reference sequences included in the mock communities at a similarity cutoff of 3%.

Greater than 80% of the experimental libraries for each of the four uneven mock communities showed taxa detection frequencies within bounds of what the theoretical sub-sampling efforts revealed (Figures 2A–2F). An exception to this was the deeper sequencing effort for bacterial mock community m2 (Figure 2B) for which two of the six replicate libraries showed lower taxa detection frequencies than would be expected by random sampling. In addition, the taxa detection frequencies for the smaller bacterial and archaeal libraries for the even communities were lower than expected. Specifically, a library size of 520±117 and 306±21 sequences resulted in taxa detection frequencies of 0.94±0.03 and 0.97±0.00 for the bacterial and archaeal mock community m1, respectively (Figures 2A and 2D). The lower than expected taxa detection frequencies for the small libraries of mock community m1 indicates that the detection of an OTU is not solely dependent on its abundance, but is also affected by the ease with which a sequence is amplified and by how susceptible it is to insertion/deletion type errors and chimera formation. For example, an approximate six to seven fold increase in sequencing depth should have improved the taxa detection frequency across all uneven mock communities. However, a significant improvement in taxa detection frequency (*p<0.05*) with greater sequencing depth was observed for only two of the four uneven mock communities. Additionally, of the 33 sequences used in the bacterial mock communities, the *S. bryantii* sequence was never detected irrespective of its relative abundance. The *S. bryantii* sequence used in these mock communities was determined to be a putative chimera with two parents and the likely position of the chimeric breakpoint was within the V3–V5 region. The non-detection of this sequence cannot be attributed to the chimera removal process used for the pyrosequencing data, since the putative chimeric sequence was included in the reference alignment used for chimera checking. Rather, it is likely that issues with amplification of this full length 16S rRNA gene that may have resulted in the formation of the putative chimera were also responsible for its poor amplification during the PCR and emPCR steps prior to pyrosequencing.

### Effect of OTU Abundance Distribution and Sequencing Depth on Mean Relative Abundance of OTUs

**Figure 3:**
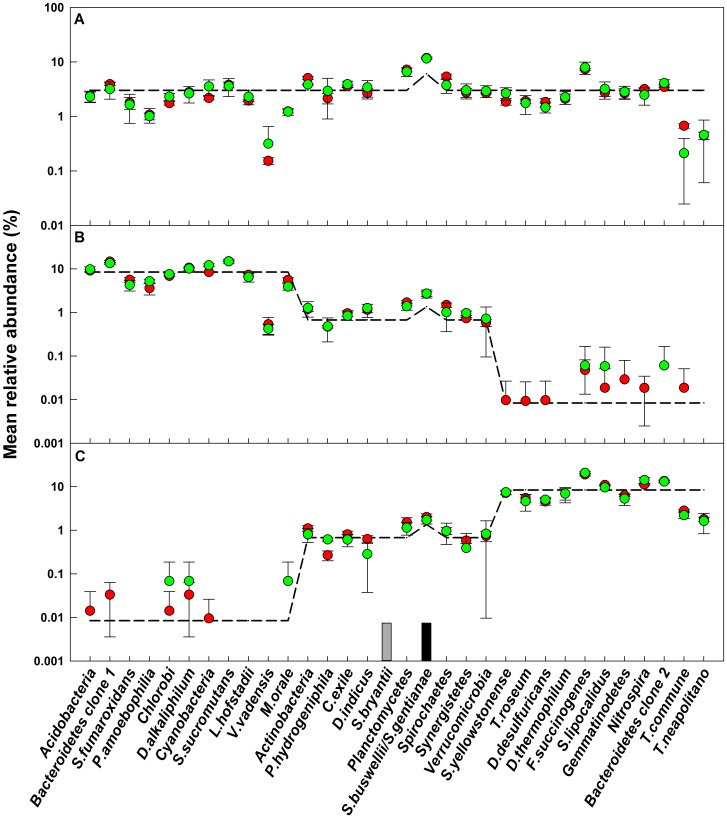
Relative abundance of sequences used to generate bacterial mock communities. A: mock1, B: mock 2, C: mock 3. Dashed line: theoretical relative abundance. The experimental mean relative abundance for small libraries (green circles) and large libraries (red circles) are shown and error bars indicate standard deviations for triplicate samples. The grey box indicates a sequence that was not detected in any community; the black box indicates an OTU that consisted of two sequences at a similarity cutoff of 3%.

**Figure 4:**
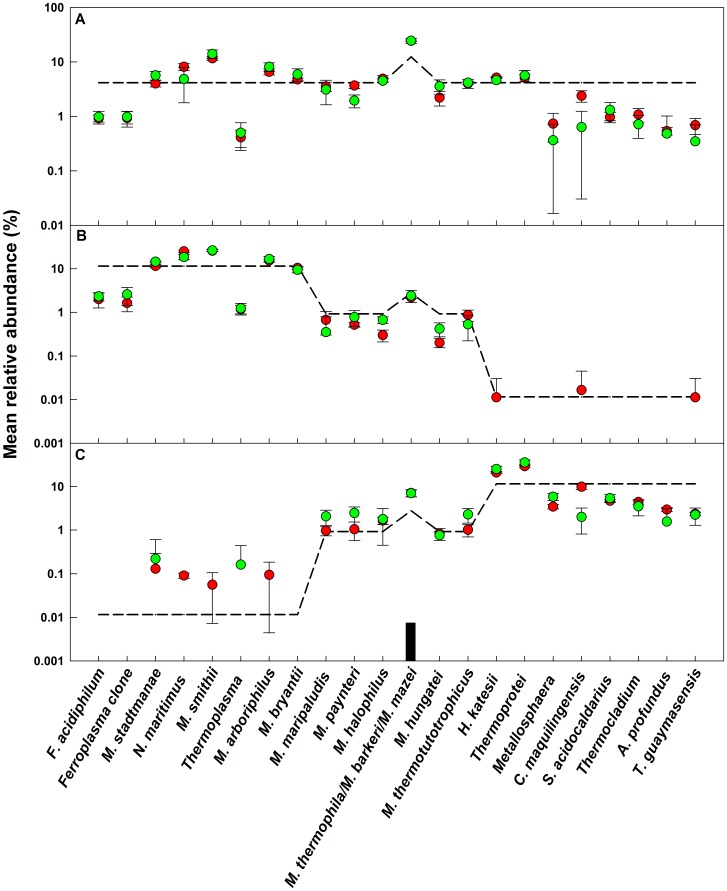
Relative abundance of sequences used to generate archaeal mock communities. A: mock1, B: mock 2, C: mock 3. Dashed line: theoretical relative abundance. The experimental mean relative abundance for small libraries (green circles) and large libraries (red circles) are shown and error bars indicate standard deviations for triplicate samples. The black box indicates an OTU that consisted of two sequences at a similarity cutoff of 3%.

The relative abundances of all sequences at a 3% similarity cutoff for bacterial and archaeal mock communities are shown in Figures 3 and 4, respectively. It is clear that experimentally determined mean relative abundance values for all OTUs showed good reproducibility between replicate libraries, across two independent sequencing runs, at two different sequencing depths, and were not affected by the differences in the number of spurious sequences in each sample library. The differences between the mean relative abundances obtained for the two runs for almost all of the OTUs were not significant (*p>0.05*). The larger sequencing depth improved the reproducibility between replicates for bacterial communities m1 and m2, and archaeal community m1, while showing no significant (*p*>0.05) reduction in variance for the other mock communities. The average experimental mean relative abundances for all sequences were 1.6±2.7 and 1.6±4.5 fold greater than the theoretical values for bacterial and archaeal mock communities, respectively. Some low abundance sequences were not detected in both, the small and large, sequencing libraries. As a result, a majority of the detected sequences were present at a higher mean relative abundance as compared to the theoretical (at the expense of the undetected OTUs), resulting in a positive error in overall mean relative abundance of all detected OTUs.

### The Effect of Errors in Mean Relative Abundance on Rank Abundance Distributions is Sample Specific

**Figure 5:**
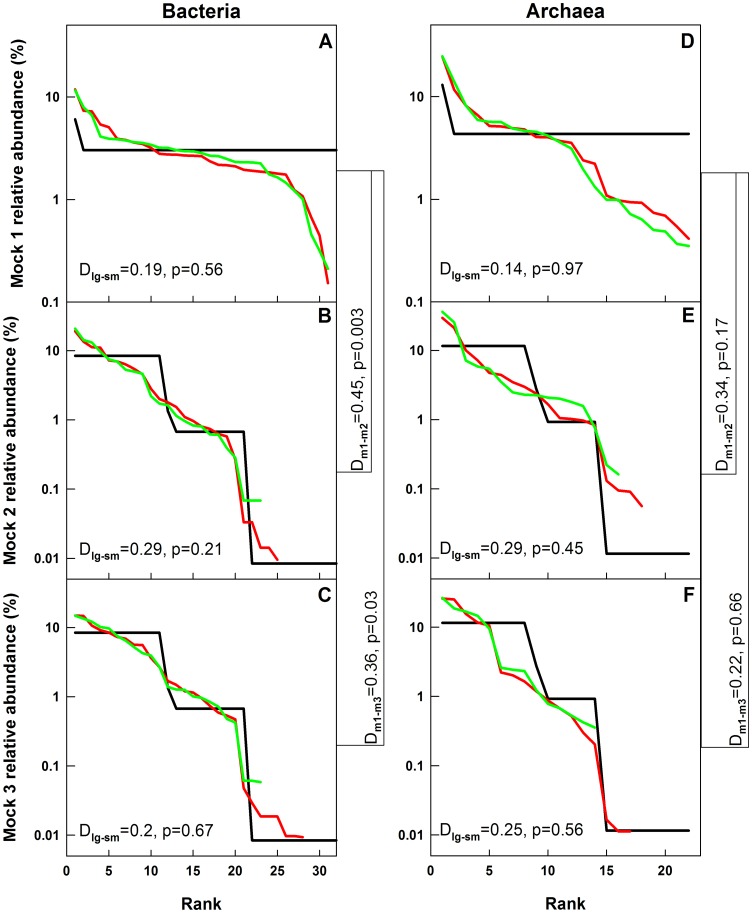
Rank abundance profiles for the bacterial and archaeal mock communities. A–C: bacteria, D–F: archaea. Black lines: theoretical, green lines: small libraries, red lines: large libraries. The Kolmogorov-Smirnov statistics at the left bottom of each panel are for comparisons between large and small libraries. The Kolmogorov-Smirnov statistics to the right of each panel are for comparisons between the large libraries of m1/m2 and m1/m3.

The experimental rank abundance distributions for all mock communities were severely distorted due to the cumulative effects of the errors in the experimentally determined mean relative abundance of each OTU (Figure 5). For this exercise, the spurious OTUs were ignored. We conducted a Kolmogorov-Smirnov test to determine if the rank abundance distributions of the even community, m1, could be distinguished from those of the two uneven communities, m2 and m3 (m2 and m3 have identical rank abundance distributions and a Kolmogorov-Smirnov test should not be able to distinguish between them). For both bacteria and archaea, the rank abundance distributions were not significantly different (*p>0.05*) (1) between the replicates of each mock community within each sequencing run, (2) for each community between two sequencing runs, and (3) for the two uneven communities (i.e., m2 and m3) (data not shown). The rank abundance distributions of bacterial m1 and m2 communities were significantly different from each other for both sequencing efforts. However, rank abundance distributions of bacterial m1 and m3 communities were only significantly different for the larger libraries (*D_large_ = 0.36, p_large_ = 0.03*). Interestingly, despite starkly different rank abundance distributions of m1 and m2/m3 mock communities, none of the experimental rank abundance distributions for the archaeal mock communities were significantly different from each other for both sampling efforts. This could be attributed to the higher quantitative error in mean relative abundance for the OTUs in the archaeal as compared to the bacterial communities (Figures 4 and 5).

### Effect of Spurious OTUs, Error in Mean Relative Abundance, and Sequencing Depth on α-Diversity

To assess the influence of the spurious OTUs, errors in mean relative abundance due to multi-template PCR biases, and sampling effort, four different α-diversity metrics were calculated. Two richness-based metrics included the number of observed OTUs (R_OBS_) and the Chao1 estimator (R_Chao1_), which utilizes R_OBS_ in combination with information about the number of singletons and doubletons in the sample library to predict the unsampled richness. Two structure-based metrics included the Inverse Simpson index (D_INVSIMP_), which calculates diversity for each community under conditions of uniform evenness, and the Non-parametric Shannon index (D_NPSHANNON_), which measures sample diversity without making any assumptions about the underlying distribution while accounting for the unsampled richness. Both D_INVSIMP_ and D_NPSHANNON_ utilize information about the number of observed OTUs and their relative abundance.

**Figure 6:**
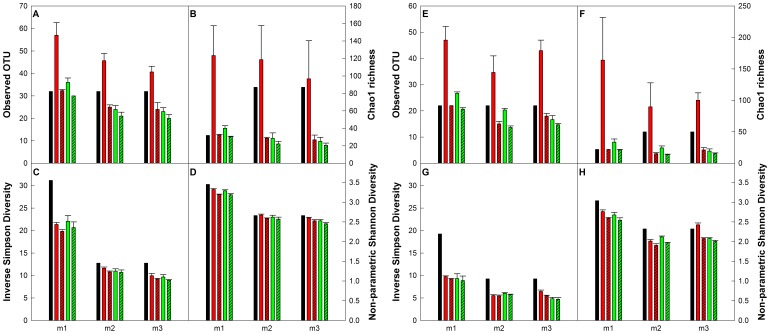
Diversity metrics calculated for the bacterial and archaeal mock communities. A–D: bacteria, E–H: archaea. Black bars: theoretical, red bars: large libraries, red-hashed bars: large libraries with spurious sequences removed, green bars: small libraries, green-hashed bars: small libraries with spurious sequences removed. The error bars indicate standard deviations for triplicate samples.

The presence of spurious OTUs resulted in significant overestimation in R_OBS_ (Figures 6A and 6E), while the predicted richness R_Chao1_ was severely inflated due to its reliance on the presence of singletons and doubletons in each library (Figures 6B and 6F). The R_Chao1_ value improved greatly for the even communities after removing the spurious OTUs, while leading to a severe underestimation of richness for the uneven communities. Additionally, the high variability (large standard deviations) in the richness estimates was due to the random presence and distribution of spurious OTUs among replicate sequence libraries for each mock community. The variance for R_Chao1_ for each community was significantly (*p<0.05*) lower when the spurious OTUs were removed. Consistent with this, the smaller libraries showed lower variability among replicates as compared to the larger libraries for both richness estimators, due to the presence of fewer spurious OTUs. Non-parametric richness estimators, such as R_Chao1_, have been reported to be highly conservative (and thus reliable) in their estimation of diversity [50]. However, our study shows that the utility of using richness estimators as a measure of α-diversity is compromised not only by the presence of spurious OTUs in pyrosequencing data, as has been noted by previous studies [51], but also due to distortions in rank abundance distributions resulting from amplification biases in multi-template PCR reactions. In contrast, the α-diversity estimators based on structure, such as D_INVSIMP_ and D_NPSHANNON_, were not significantly (*p>0.05*) affected by the presence of spurious OTUs, the errors in mean relative abundance, and differences in sequencing depth between the two sequencing runs (Figures 6C, 6D, 6G, 6H). Even though D_INVSIMP_ estimates were lower than theoretical values for all mock communities, they still maintained the theoretical trend in diversity, i.e., m1>m2, m3. Additionally, D_NPSHANNON_ values were very similar to the theoretical estimates for all the mock communities, even with the smaller sequencing effort, and maintained the theoretical trend in diversity similar to D_INVSIMP_ estimates. These results highlight the usefulness of the structure-based over richness-based α-diversity metrics for pyrosequencing studies.

### Effect of Spurious OTUs, Error in Mean Relative Abundance, and Sampling Effort on Deviation from Theoretical Community Structure

**Figure 7:**
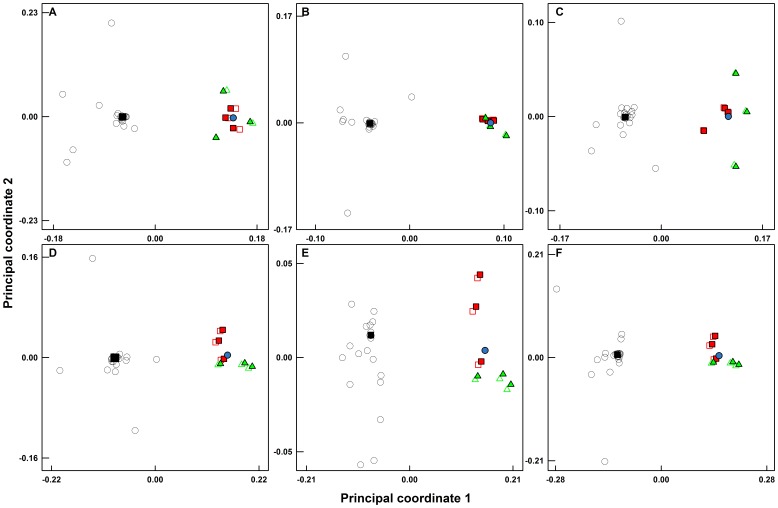
Principal coordinate axes plot for bacterial and archaeal communities constructed using the Morisita-Horn distance (D_MH_). A–C: bacteria, D–F: archaea. A/D: m1, B/E: m2, C/F: m3. Black squares indicate the theoretical mock community and the small open circles denote the *in-silico* sequencing efforts at sampling depths varying from 1 to 90%. The red filled squares and red open squares represent the large libraries with and without spurious OTUs, respectively, while green filled triangles and green open triangles indicate the small libraries with and without spurious OTUs, respectively. The blue circle is the centroid of the experimental libraries.

Figure 7 shows principal coordinate analyses plots using the Morisita-Horn distance (D_MH_ distance) for the bacterial and archaeal mock communities at two different sequencing depths and with and without the inclusion of spurious OTUs. While the sub-sampling efforts of the *in-silico* communities converged onto the theoretical position with increasing sequencing depth, both the bacterial and archaeal experimental libraries clustered independently from the theoretical communities. Additionally, the removal of spurious OTUs from each library did not result in any significant movement towards the theoretical position. To further assess the benefits of greater sequencing depth and removal of spurious OTUs, we compared the D_MH_ distances between the experimental sequencing libraries and the theoretical communities (Figure S3). A significant improvement with increased sequencing depth, i.e., lower distance from theoretical, was only seen for three of the six mock communities, specifically bacteria m1 (*p = 0.003*), bacteria m2 (*p<0.0001*), and archaea m3 (*p<0.0001*), despite having six to seven fold more sequences in the larger libraries as compared to the smaller libraries. Hence, it is clear that the benefits of greater sequencing depth in presenting a more accurate picture of the sampled community were dependent on the OTUs present and their relative abundance in any given sample. However, the larger sequencing libraries were less susceptible to the presence of spurious OTUs compared to the smaller sequencing libraries (Figure S3), specifically bacteria m1 (*p = 0.0001*), bacteria m2 (*p = 0.0007*), and archaea m1 (*p<0.0001*) communities. This shows that, even though a greater sequencing depth may not provide a more accurate representation of all sampled communities, larger libraries may be less affected by the presence of spurious OTUs than smaller libraries.

### Effect of Spurious OTUs, Error in Mean Relative Abundance, and Sampling Effort on β-Diversity

**Figure 8:**
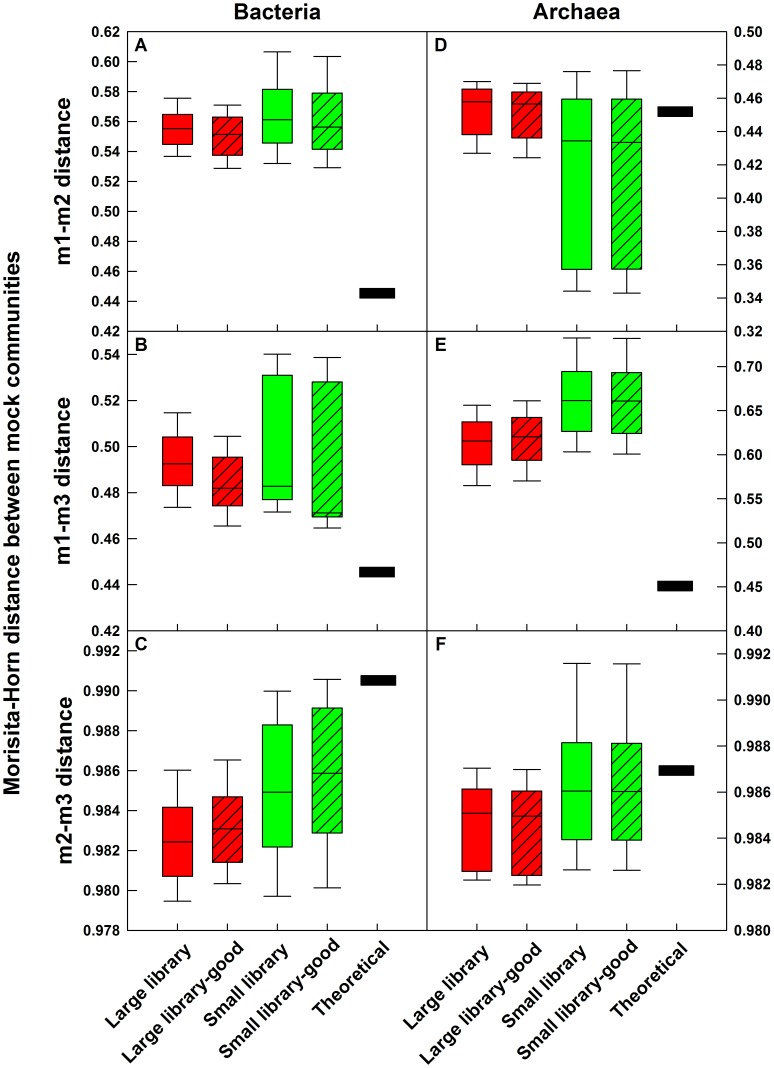
Comparisons of pairwise Morisita-Horn distance between bacterial and archaeal mock communities to the theoretical pairwise distances. A–C: bacteria, D–F: archaea. A/D: m1–m2, B/E: m1–m3, C/F: m2–m3. Nine pairwise comparisons generated between three replicate libraries for each community were used to construct each box.

We calculated Jaccard (D_JACCARD_) and D_MH_ distances between the experimental libraries and compared them to the distances between the theoretical communities. D_JACCARD_ is a shared-richness based metric, which utilizes a presence/absence approach and estimates the distance between two samples based on the number of OTUs unique to each sample. In contrast, D_MH_ is a shared-structure based metric and utilizes the relative abundance of each shared and unique OTUs while estimating the distance between two samples. The theoretical D_MH_ distances between m1 and m2/m3 were 0.444 and 0.451, while the theoretical D_MH_ distances between m2 and m3 were 0.991 and 0.987, for bacterial and archaeal mock communities, respectively. Figure 8 provides a comparison between the experimentally determined pairwise D_MH_ distances and the theoretical values. The D_MH_ for the larger and smaller sequencing libraries were not significantly different, indicating that deeper sequencing did not result in improved accuracy (i.e., reduction in difference between experimental and theoretical estimates) in β-diversity estimates for both bacterial or archaeal communities. However, the variance between replicates was significantly lower for the larger libraries as compared to the smaller libraries (F-test, *p<0.05*), indicating improved precision with deeper sequencing. Further, removal of spurious sequences from both the large and the small libraries also did not improve the D_MH_ accuracy, indicating that the left over spurious sequences had minimal impact on β-diversity. The lack of improved accuracy by either deeper sequencing effort or spurious OTU removal clearly indicates that the errors in mean relative abundance resulting from multi-template PCR bias play a significant role in limiting the accuracy of β-diversity estimates calculated using structure based metrics.

Similar to α-diversity metrics, β-diversity measures that rely on shared richness were highly vulnerable to both sampling effort and the presence of spurious OTUs. For example, the D_JACCARD_ distance for m1–m2, m1–m3, and m2–m3 comparisons of the theoretical communities is 0, since they have identical sequences and the overlap between the three communities is perfect. However, the pairwise D_JACCARD_ distances between archaeal m1–m2, m1–m3, and m2–m3 without the spurious OTUs were 0.33±0.04, 0.27±0.03, and 0.62±0.06, respectively. The ∼30% difference between archaeal m1–m2 and m1–m3 is due to the fact that 33% of the low abundance OTUs in each of the uneven communities were not reliably detected in each library. The difference between m2–m3 was approximately 60% since the high abundance OTUs in m2 were the undetected low abundance OTUs in m3 and *vice versa*. When all OTUs were considered, including the spurious OTUs, the pairwise D_JACCARD_ distances between m1–m2, m1–m3, and m2–m3 were 0.75±0.03, 0.66±0.04, and 0.85±0.01, respectively. It is clear that the spurious OTUs had a significant effect on the β-diversity comparisons between the three mock communities while using the Jaccard index. The challenges associated with richness-based metrics are more complicated for environmental samples, in which rare taxa are expected to be detected only sporadically in replicate sequencing libraries of the same sample [51] and will be indistinguishable from the spurious OTUs.

### Summarizing the Contributions of this Study

Amplicon-based pyrosequencing methods have major advantages over the tools that have been extensively used in the past to study microbial community structure. They offer multiplexing capabilities similar or greater than those provided by common DNA fingerprinting tools [52], [53], while also delivering DNA sequence information to identify taxa and relative abundance values. However, it is important to consider how advanced sequencing tools can be reliably applied to study environmental systems while minimizing the effects of their current limitations. To this end, our study provides several recommendations for the appropriate use of pyrosequencing data to study microbial communities.

First, we recommend that richness and shared richness metrics should not be used to draw quantitative conclusions about α- and β-diversity based on pyrosequencing data. These metrics are significantly affected by the presence of sequencing errors and variation in sequencing depth between samples (Figure 6A, B, E, and F). Second, we show that structure-based metrics that utilize richness and relative abundance of each OTU are less affected by sequencing depth and errors (Figure 6C, D, G, and H) and can be used reliably to draw quantitative conclusions about the sequenced communities. Third, we show that greater sequencing depth does not always result in a more accurate representation of the sequenced community (Figure 7), since the errors in mean relative abundance due to multi-template PCR bias significantly alter the rank abundance distributions (Figure 5). In fact, in most situations, the error in mean relative abundance is the primary impediment to understanding microbial community structure (Figure 5). Fourth, we show that the benefit of greater sequencing depth lies in the improved precision of structure based β-diversity estimates by reducing variability between replicates and that deeper sequencing does not necessarily improve accuracy of β-diversity estimates. A fifth contribution of this study is the observation that the number of sequences in each sample library may be affected by the GC content of the amplicon pool of each sample (inter-sample bias). Based on this finding, we encourage future studies to investigate the extent to which GC bias affects the relative abundances of OTUs (intra-sample bias). Finally, we have tested and made available updated designs of bacterial and archaeal 454-compatible primers along with multiplexing barcodes. These primers show high database coverage of most bacterial and archaeal phyla and allow for simultaneous multiplexing of separate bacterial and archaeal amplicon pools from the V3–V5 region of the 16S rRNA gene.

## Materials and Methods

### Primer and Multiplexing Barcode Design

Primers targeting the V3–V5 region of the bacterial and archaeal 16S rRNA gene were modified from previously published primers [34], [36], [37], [54] (Tables 1 and S1). The coverage of old and new primer sets was checked against good quality sequences (as defined based on Pintail scores by the RDP database) greater than 1,200 bp in the RDP database (Release 10) using the feature Probematch (Figure S1 and Table 1) [55]. Multiplexing barcodes were designed to allow for simultaneous sequencing of bacterial and archaeal amplicons using Barcode Designer Software (http://sourceforge.net/projects/jcvibard/). Information about design criteria, barcode sequence, primer interactions, and secondary structure potential is provided in Table S1. All primers were synthesized and HPLC purified by Integrated DNA technologies (Coralville, IA).

### Mock Community Preparation

A total of 33 bacterial 16S rRNA gene sequences belonging to 27 different phyla and 24 archaeal 16S rRNA gene sequences belonging to three different phyla were used to construct two sets of three mock communities, all of which provided target regions with perfect matches to the bacterial and archaeal primers designed in this study, respectively. Of the 57 sequences used, ten bacterial and five archaeal sequences were clones originating from environmental samples, while the remaining sequences were obtained from pure cultures of bacteria and archaea (Table S4). Clones of near full length 16S rRNA gene fragments were generated using PGEM-T Easy Vector II system (Promega Inc, Madison, WI) according to manufacturer specifications. Information about primers used to amplify near full length sequences are provided in Table S6. Plasmids containing cloned inserts were sequenced at the University of Michigan DNA sequencing core (Ann Arbor, MI) and the sequences were deposited in Genbank (accession numbers: JQ346727–JQ346782). Even though all sequences included in the mock communities were checked for chimeras, one bacterial sequence (*S. bryantii*) was at the end of the study determined to be a putative chimera and hence was not deposited in GenBank. The plasmid concentrations were quantified in triplicate using Quant-iT dsDNA assay kit (Invitrogen, Carlsbad, CA) and quantified on a Nanodrop 3300 (Thermo Scientific, Wilmington, DE). Following quantitation of plasmid concentrations, two sets of three mock communities were constructed as follows. The sequences were first divided into three clusters each for bacteria and archaea based on the GC content (mean % GC content±standard deviation) of the V3–V5 region (Figure S2), i.e., low GC (bacteria: 51.1±2.1, archaea: 53.1±1.9), medium GC (bacteria: 55.1±1.8, archaea: 58.3±0.5), and high GC (bacteria: 59.3±3.3, archaea: 64.9±2.1) with 11 bacterial and eight archaeal sequences in each cluster. The two sets of mock communities consisted of mock1 (m1) (all sequences with equal abundance), and two uneven communities, mock2 (m2) and mock3 (m3). The four uneven communities were designed to have three abundance levels with one GC cluster at each abundance level. The relative abundance of each sequence in the three bacterial and archaeal mock communities is provided in Figure S2. Each mock community was prepared three times by independently mixing each sequence, to generate technical replicates.

### Environmental Samples

The PCR conditions were identical to those used for the mock community samples and are detailed below. The OP-YNP, MGCT, and GC samples were donated by M. Podar (Oak Ridge National Laboratories), V. Young (University of Michigan), and G. Dick (University of Michigan), respectively. The SW sample was collected from the Huron River in Ann Arbor, Michigan, while DWDS sample was collected from a local drinking water distribution system [56]. The ANBR and FAS samples were collected from a laboratory scale anaerobic bioreactor used to treat low strength wastewaters at the University of Michigan and from a fresh water aquaculture system located in Milwaukee, Wisconsin.

### PCR Amplification and Sample Preparation for Sequencing

The GC content of Bact-909R and Arch-915R primers with the multiplexing barcodes and fusion primers varied between 47.8–57.2% (52.5±1.9%, *n = 70*) and 58.5–66% (62.4±1.6%, *n = 85*) (Table S1), respectively. The differences in multiplexing barcode sequences altered the secondary structure formation potential for each reverse primer. To account for any variability between replicates due to differences in reverse primer characteristics, each replicate of the mock communities was subjected to PCR with primers that exhibited significantly different GC contents. Specifically, the first (m1.1, m2.1, m3.1), second (m1.2, m2.2, m3.2), and third (m1.3, m2.3, m3.3) replicates of each mock community were subjected to PCR with reverse primer with a GC content of 47.8, 51.6, and 55.3% for bacterial communities and 60.4, 62.3, and 64.3% for archaeal communities, respectively. The amplification of replicate mock communities with reverse primers with varying GC fractions resulted in amplicon pools corresponding to each replicate with slightly different GC contents (Figure 1A). The PCR reactions were conducted in triplicate [26] and were limited to 15 cycles to minimize formation of PCR artifacts [57]. The plasmids containing 16S rRNA inserts were not linearized prior to PCR, which may have resulted in slight variations in PCR amplification efficiency between different sequences in the mock communities. Each PCR reaction mix contained 10 µl of *PfuUltraII* hotstart mastermix (Stratagene, Santa Clara, CA), 0.2 µM of equimolar mix of the forward primers (if more than one was used), 0.2 µM reverse primer, 0.3 mg/ml of bovine serum albumin (Invitrogen, Carlsbad, CA), a final DNA template concentration of 4 ng/µl of DNA, and PCR grade water to a total volume of 20 µl. The PCR thermocycling conditions were as follows: 2 min at 95°C, and 15 cycles of 95°C for 20 s, 50/55°C (bacteria/archaea) for 20 s, 72°C for 30 s, followed by a final extension at 72°C for 3 min. The thermocycling conditions and DNA template and primer concentrations were optimized to maximize yield of PCR product in 15 cycles (data not shown).

Following PCR amplification, the triplicate PCR reactions for each sample preparation were pooled and purified. The amount of PCR product from each sample was quantified in triplicate using Quant-iT dsDNA assay kit (Invitrogen, Carlsbad, CA) on a Nanodrop 3300 (Thermo Scientific, Wilmington, DE). Two different PCR product pools were generated, one each for bacteria and archaea. Each pool had equal amounts of PCR product originating from either mock community or environmental sample. Both PCR product pools were then run on 2% agarose gel at 50 Volts for 60 min. The bands corresponding to 600–700 bp for bacteria and 600–900 bp for archaea were excised and purified using a Qiaquick Gel Extraction kit (Qiagen, Valencia, CA). A larger range of amplicon sizes was extracted for archaea as the V3–V5 region of *C. maquilingensis* is 733 bp in size. Following gel extraction, both PCR product pools were re-purified using a Qiaquick PCR purification kit (Qiagen, Valencia, CA). The purified archaeal and bacterial amplicon pools were quantified as described above, and were mixed in 60∶40 (bacteria:archaea) proportions to generate the final amplicon pool and sent for 454-titanium sequencing.

### 454-titanium Sequencing

Amplicon pools of the mock communities were sequenced on two separate occasions at two different sequencing facilities. For both runs, sequencing was performed from the V5 to the V3 region. The first sequencing run included the 18 mock community samples combined with 60 environmental samples and was performed at the Michigan State University Research Technology Support Facility (East Lansing, Michigan) on 1/8^th^ pico-titer plate. This run yielded 30,910 reads with a pass rate of 20% giving 300–600 reads per sample after quality filtering (detailed below). The second sequencing run included the 18 mock community samples and 12 environmental samples and was conducted at the University of South Carolina Environmental Genomic Core Facility (Columbia, SC) on 1/8^th^ pico-titer plate. This run yielded 73,403 sequences with a pass rate of 37% and provided between 2,000–3,000 sequences per sample after quality filtering (described below). The observed pass rates, between 20–40%, are expected for amplicons longer than 400 bp on the 454 sequencing platform as compared to 50–60% pass rates for amplicons shorter than 400 bp [58].

### Sequence Data Processing and Analyses

All data processing was conducted using Mothur [59]. We did not utilize Denoising protocols [18]–[20] while processing the pyrosequencing output in this study. Denoising protocols employ sequence correction approaches to reduce the level of noise in pyrosequencing data originating from the sequencing process and PCR amplification. Though denoising generally is an essential step towards processing pyrosequencing datasets, the goal of this study was not to correct the noise, but assess its impact on the interpretation of the sampled community structure and compare it to other factors such as sequencing depth and errors in mean relative abundance of OTUs. Additionally, the mock communities in this study were composed of known sequences. Hence, we were able to identify spurious sequences and selectively remove them from the experimental dataset and assess the changes in community structure and membership resulting from their removal. As a result, we did not use denoising protocols, but a simple yet stringent quality filtering protocol (detailed below) to ensure removal of sequencing noise at a defined quality control threshold. Specifically, the sequences were quality filtered to allow a maximum of 1 bp mismatch with the reverse primer, 0 mismatches with the barcode, 0 ambiguous bases, and an average quality score (q_average_) of 25 over a sliding window of 50 bp over the read length. The 1 bp mismatch with the primer was allowed since the primer region is not used for any subsequent analyses and this allows for retention of sequences that may otherwise be good. However, we did not allow any mismatches with the barcodes since they are used for sample sorting and may affect how sequences are binned. If the q_average_ over the defined sliding window dropped below 25, the distal end of the read was trimmed and only the sequencing end was retained. Following this, all reads that were quality trimmed below 200 bp were removed from the library. The remaining sequences were aligned against a custom seed alignment with *k-mer* searching using a k-size of 8.0 and Needleman-Wunsch pairwise alignment. The seed alignment for the mock communities was generated by aligning near-full length reference sequences used in the mock communities using the SINA-aligner [60]. Sequences whose alignment did not terminate at the V5 region were removed as poorly aligned sequences. Subsequently, the remaining sequences were checked for the presence of chimeras by comparing against the same seed alignment using the UCHIME algorithm [21] in Mothur and any sequences flagged as chimeras were removed. All the reads retained after the chimera removal step were considered quality filtered reads. Information about the number of quality filtered and chimera-free reads in each mock community sample are provided in Table S5.

Next, two alignments each consisting of three *in-silico* bacterial or archaeal mock communities were merged with the bacterial or archaeal experimental alignments, respectively. The *in-silico* mock communities were generated by combining all sequences in the same relative abundances as shown in Figure S2. This was done so the lowest abundance sequences had one read each in m2 and m3. The medium and high abundance sequences were then adjusted accordingly to yield final bacterial and archaeal *in-silico* mock communities with 11,891 and 8,640 reads, respectively. The size of the even mock community, m1, was increased to match the total reads in the respective uneven mock communities. After merging the alignments for experimental and *in-silico* mock communities, the resulting alignment was filtered using the vertical = T and trump = ., options in Mothur. This ensures that sequences are compared along similar parts of the 16S rRNA gene, while calculating the distance matrix [16]. The resulting filtered alignment was 388 and 603 columns for bacteria and archaea, respectively. A distance matrix was generated in PHYLIP format [61], and the sequences were clustered into OTUs using the average neighbor method [18].

All the experimental reads that clustered with one of the reference sequences at a similarity cutoff of 3% [17] were categorized as “good” sequences, while the others were tagged as spurious sequences. The relative abundance of each OTU was estimated based on the percent reads in each sample library clustering with the respective reference sequence at a similarity cutoff of 3%. To separate the effects of sequencing depth and errors in mean relative abundance from that of spurious OTUs on community structure evaluations, all the spurious sequences were removed and the libraries with only good sequences, referred to as “good libraries”, were analyzed alongside the libraries with all the sequences included and the *in-silico* mock communities. Additionally, to assess the effect of sequencing depth alone, the *in-silico* bacterial and archaeal sequence libraries were randomly sub-sampled in triplicate to generate three sub-sample libraries each, containing 1, 5, 10, 20, 50, 70, and 90% of the sequences in the original *in-silico* libraries. These sub-sample libraries were analyzed alongside the experimental libraries with and without spurious sequences and the complete *in-silico* libraries. The entire workflow is also presented in Figure S4.

### Diversity, Classification and Similarity Estimates and their Statistical Significance

The environmental sequence libraries were classified using the classification seed files provided through Mothur and using the k-nearest neighbor approach and a cutoff of 80%. If an expected class was not detected in a sample, then the presence/absence confirmation for this class was further conducted using specific primers available in the literature as discussed in the Results and Discussion section. For all experimental mock community libraries (with and without spurious sequences) and *in-silico* libraries (full and sub-sample), α- and β-diversity metrics based on the OTU-based approach were estimated using Mothur. The α-diversity metrics included the number of observed OTUs (R_OBS_), the Chao1 estimator (R_chao1_), Inverse-Simpson (D_INVSIMP_) metric, and non-parametric Shannon (D_NPSHANNON_) metric. The Morisita-Horn similarity index (D_MH_) and Jaccard Index (D_JACCARD_) were used to calculate distance between samples to evaluate β-diversity. Microsoft Excel and SPSS statistical package were used for statistical analyses not provided through the Mothur platform. The mean relative abundance of OTUs were compared using the two-tailed student t-test without making assumptions about the variances (α = 0.05). The non-parametric Kolmogorov-Smirnov test was used to determine whether the experimental rank abundance distributions generated at different sampling efforts and community structures were significantly different. The F-test was used to compare variances of diversity and pairwise distance estimates across replicate samples at different sampling efforts with and without spurious sequences (α = 0.05).

## Supporting Information

Figure S1 Coverage of the newly designed primers (red-Table S1) and previously used primers (blue-Table S8-Bact-338F old+Bact-909R old, Arch-340F+Arch-934R) [1]–[3] targeting the V3–V5 hypervariable regions of (A) bacterial and (B) archaeal 16S rRNA genes. Coverage for *Crenarchaeota* and *Euryarchaeota* is shown at the order-level, while other bacterial and archaeal coverage is shown at the phylum level. Coverage was checked by using the probe match function against the RDP database (Release 10) for good quality sequences greater than 1,200 bp and allowing 0 mismatches [4]. Note that the phylum *Thaumarchaeota* does not contain any sequences in the RDP database. However, sequences from the *Nitrosopumilaceae* family (proposed member of *Thaumarchaeota* [5]) were detected in environmental samples analyzed in this study and are therefore included in this analysis. Additionally, even though the new and old archaeal primer sets do not target *Nanoarchaeota*, sequences identified as *Nanoarchaeota* were detected in environmental samples tested in this study.
(DOC)

Figure S2 The relative abundance of the 33 and 24 sequences used in the (A) bacterial and (B) archaeal mock communities. Solid line: m1, dotted line: m2, dashed line: m3. The relative abundance values (%) corresponding to each abundance level are shown next to the plots.
(DOC)

Figure S3 Morisita-Horn distance (D_MH_) between experimental libraries and theoretical communities for bacterial (A, B, C) and archaeal (D, E, F) mock communities. Panels A and D indicate the effect of sequencing depth on D_MH_ (red bars: large libraries, green bars: small libraries), Panels B/E and C/F indicate the influence of removal of spurious OTUs on D_MH_ for the large libraries (red bars: large libraries, red-hashed bards: large libraries-spurious OTUs removed) and small libraries (green bars: small libraries, green-hashed bars: small libraries-spurious OTUs removed), respectively. The stars indicate D_MH_ values that were significantly different (*p<0.05*).
(DOC)

Figure S4 Schematic showing workflow from the raw sequencing libraries up to the generation of the “working file” of sequences used for all the results presented in this study. Details for each step are presented in the materials and methods section.
(DOC)

Table S1 Sequence information for template specific bacterial and archaeal primers, multiplexing barcodes, and thermodynamic and secondary structure parameters. (A) Sequence information for the three bacterial forward primers, Bact-338F1, Bact-338F2, and Bact-338F3, and thermodynamic parameters for the primers when used in combination with Titanium fusion primer B. (B) Sequence information for the reverse bacterial primer Bact-909R and designed barcodes. Also provided are the thermodynamic parameters for the most stable hairpin, homo-dimer, and hetero-dimer interactions with the three forward primers. (C) Sequence information for the archaeal forward primer, Arch-340F, and thermodynamic parameters for the primer when used in combination with Titanium fusion primer B. (D) Sequence information for the reverse archaeal primer Arch-915R and designed barcodes. Also provided are the thermodynamic parameters for the most stable hairpin, homo-dimer, and hetero-dimer interactions with the forward primer. Units for thermodynamic parameters: dG = kcal/mole, dH = kcal/mole, dS = cal/mole.K, Tm = °C. The barcodes were designed with the following constraints: (1) ten nucleotides in length, (2) maximum of five flows for complete resolution, and (3) a minimum Levenshtein distance of 3 between any two barcodes. The fusion primer-barcode-template primer combinations were screened for potential for formation of homo-dimers, hetero-dimers, and hairpin structures using dinamelt as a primary screening tool followed by the Oligoanalyzer tool available through IDT-DNA (http://www.idtdna.com/analyzer/applications/oligoanalyzer/) and only primers that were free from potential secondary structure issues were retained.
(XLS)

Table S2 The detection of bacterial sequences in environmental samples at resolution down to the order level. The classification was conducted on quality filtered and chimera free sequence libraries for each sample. Green boxes indicate sequence classes that were detected.
(PDF)

Table S3 The detection of archaeal sequences in environmental samples at resolution down to the family level. The classification was conducted on quality filtered and chimera free sequence libraries for each sample. Green boxes indicate sequence classes that were detected.
(PDF)

Table S4 Information about the source of each sequence, the length of near full-length 16S rRNA gene sequences amplified, the length of the V3–V5 region, and the GC content and length of the longest homopolymer in the full length and V3–V5 region.
(DOC)

Table S5 The number of sequences obtained for each bacterial and archaeal mock community before and after quality filtering and chimera removal. Sm- small library generated during first sequencing run, lg-large library generated during second sequencing run.
(DOC)

Table S6 Primers used to amplify near full-length 16S rRNA gene sequences used to generate the mock communities.
(DOC)
