# Supplementary material for: PCR Biases Distort Bacterial and Archaeal Community Structure in Pyrosequencing Datasets
Source: PLoS One. 2012 Aug 15;7(8):e43093. doi: 10.1371/journal.pone.0043093 (PMC3419673; doi:10.1371/journal.pone.0043093)
Supplement: Figure S1 — Coverage of the newly designed primers (red-Table S1) and previously used primers (blue-Table S8-Bact-338F old+Bact-909R old, Arch-340F+Arch-934R) [1]–[3] targeting the V3–V5 hypervariable regions of (A) bacterial and (B) archaeal 16S rRNA genes. Coverage for Crenarchaeota and Euryarchaeota is shown at the order-level, while other bacterial and archaeal coverage is shown at the phylum level. Coverage was checked by using the probe match function against the RDP database (Release 10) for good quality sequences greater than 1,200 bp and allowing 0 mismatches [4]. Note that the phylum Thaumarchaeota does not contain any sequences in the RDP database. However, sequences from the Nitrosopumilaceae family (proposed member of Thaumarchaeota [5]) were detected in environmental samples analyzed in this study and are therefore included in this analysis. Additionally, even though the new and old archaeal primer sets do not target Nanoarchaeota, sequences identified as Nanoarchaeota were detected in environmental samples tested in this study. (DOC) [file pone.0043093.s001.doc]

**Figure S1**. Coverage of the newly designed primers (red-Table S1) and previously used primers (blue-Table S8-Bact-338F old+Bact-909R old, Arch-340F+Arch-934R) [1-3] targeting the V3-V5 hypervariable regions of (A) bacterial and (B) archaeal 16S rRNA genes. Coverage for *Crenarchaeota* and *Euryarchaeota* is shown at the order-level, while other bacterial and archaeal coverage is shown at the phylum level. Coverage was checked by using the probe match function against the RDP database (Release 10) for good quality sequences greater than 1,200 bp and allowing 0 mismatches [4]. Note that the phylum *Thaumarchaeota* does not contain any sequences in the RDP database. However, sequences from the *Nitrosopumilaceae* family (proposed member of *Thaumarchaeota* [5]) were detected in environmental samples analyzed in this study and are therefore included in this analysis. Additionally, even though the new and old archaeal primer sets do not target *Nanoarchaeota*, sequences identified as *Nanoarchaeota* were detected in environmental samples tested in this study.


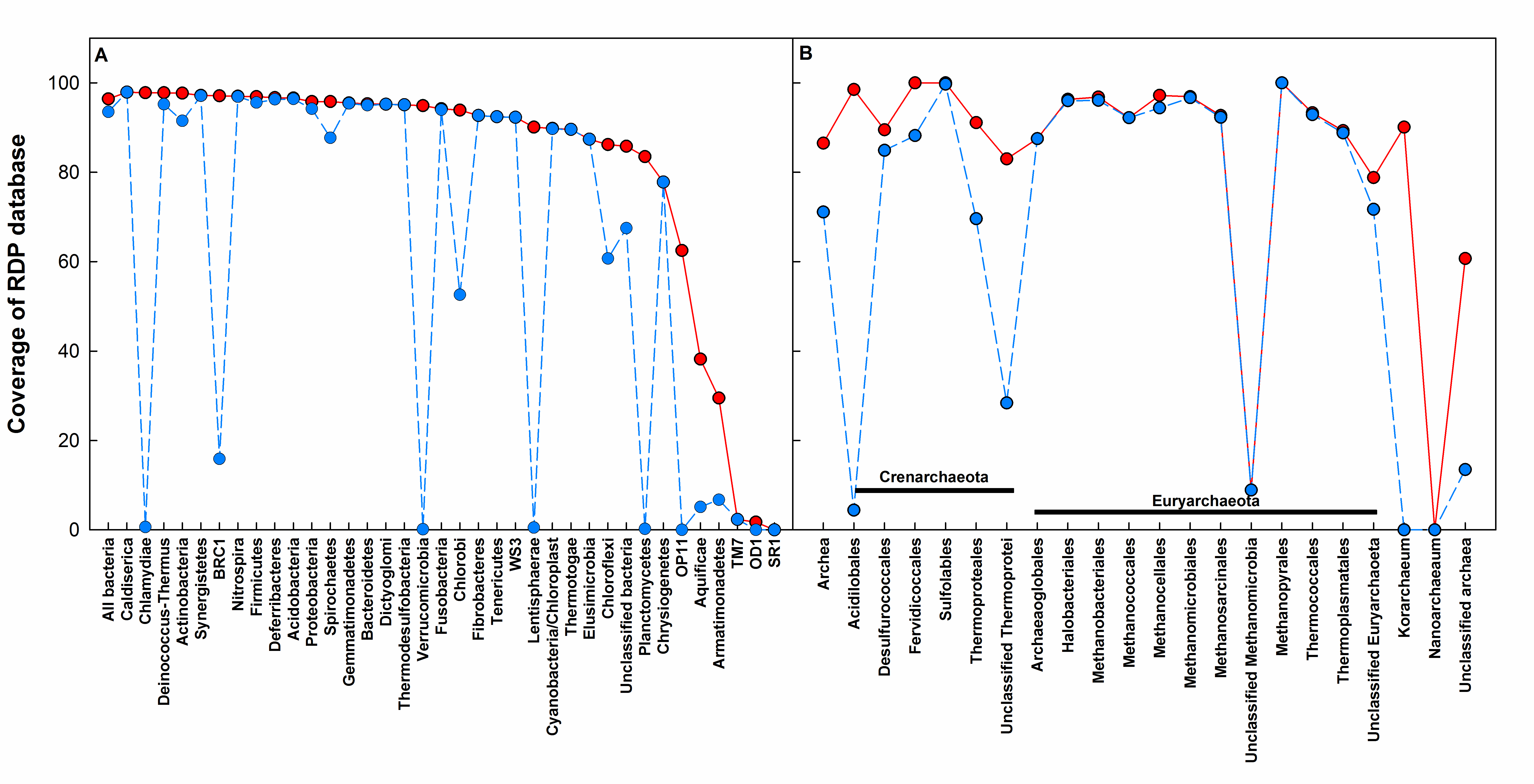


1. Haas B, Gevers D, Earl A, Feldgarden M, Ward D, et al. (2011) Chimeric 16S rRNA sequence formation and detection in Sanger and 454-pyrosequenced PCR amplicons. Genome Res 21: 494-504.

2. Stahl DA, Amann R (1991) Development and application of nucleic acid probes. In: Stackebrandt E, Goodfellow M, editors. Nucleic acid techniques in bacterial systematics. Chichester, England: John Wiley & Sons. pp. 205-248.

3. Baker G, Smith J, Cowan D (2003) Review and re-analysis of domain-specific 16S primers. J Microbiol Methods 55: 541-555.

4. Cole J, Wang Q, Cardenas E, Fish J, Chai B, et al. (2009) The Ribosomal Database Project: improved alignments and new tools for rRNA analysis. Nucl Acids Res 37: D141-145.

5. Spang A, Hatzenpichler R, Brochier-Armanet C, Rattei T, Tischler P, et al. (2010) Distinct gene set in two different lineages of ammonia-oxidizing archaea supports the phylum *Thaumarchaeota*. Trend Microbiol 18: 331-340.
