# Supplementary material for: PCR Biases Distort Bacterial and Archaeal Community Structure in Pyrosequencing Datasets
Source: PLoS One. 2012 Aug 15;7(8):e43093. doi: 10.1371/journal.pone.0043093 (PMC3419673; doi:10.1371/journal.pone.0043093)

**Figure S2**. The relative abundance of the 33 and 24 sequences used in the (A) bacterial and (B) archaeal mock communities. Solid line: m1, dotted line: m2, dashed line: m3. The relative abundance values (%) corresponding to each abundance level are shown next to the plots.


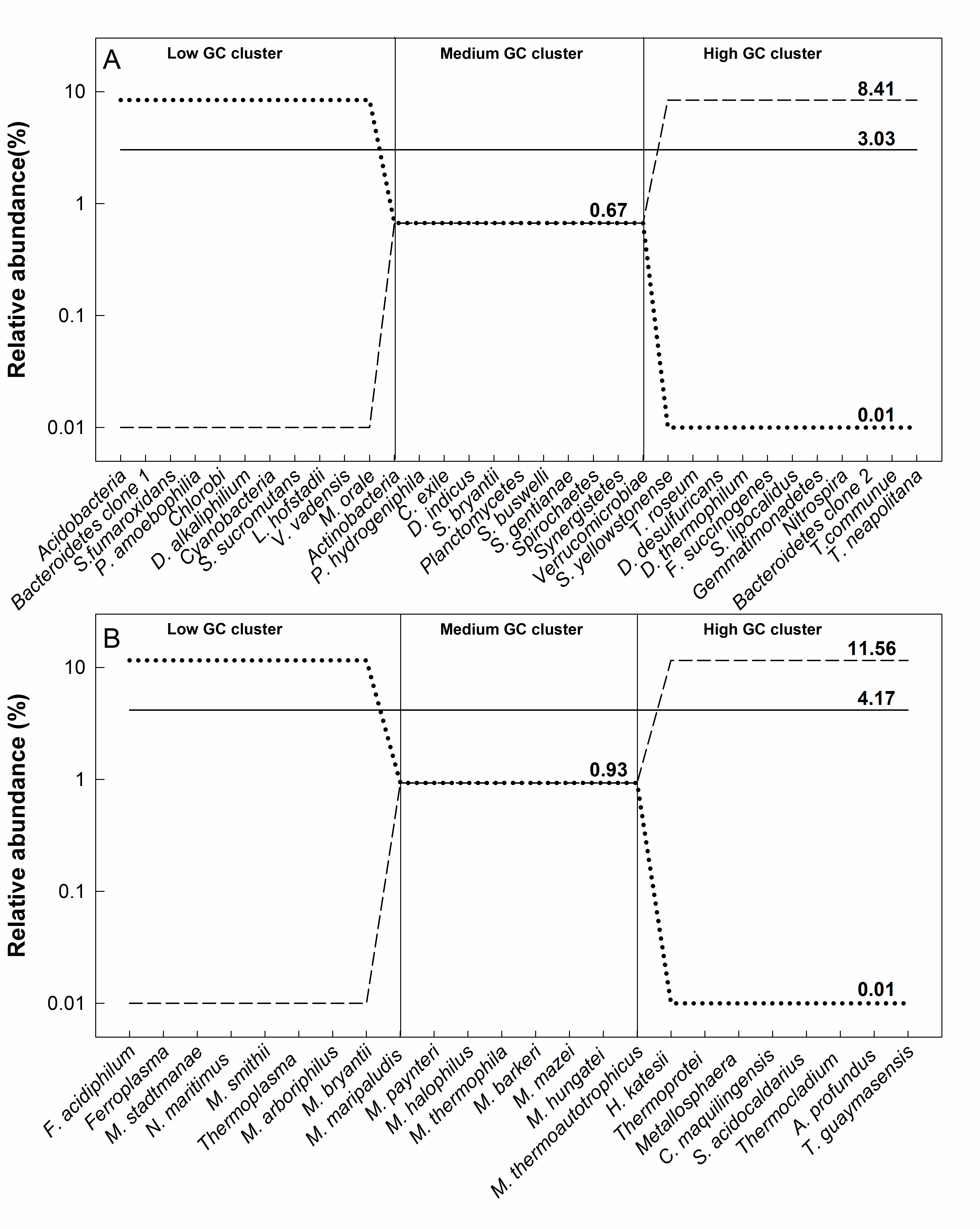

Supplement: Figure S2 — The relative abundance of the 33 and 24 sequences used in the (A) bacterial and (B) archaeal mock communities. Solid line: m1, dotted line: m2, dashed line: m3. The relative abundance values (%) corresponding to each abundance level are shown next to the plots. (DOC) [file pone.0043093.s002.doc]
