# Supplementary material for: PCR Biases Distort Bacterial and Archaeal Community Structure in Pyrosequencing Datasets
Source: PLoS One. 2012 Aug 15;7(8):e43093. doi: 10.1371/journal.pone.0043093 (PMC3419673; doi:10.1371/journal.pone.0043093)

**Figure S3.** Morisita-Horn distance (D_MH_) between experimental libraries and theoretical communities for bacterial (A, B, C) and archaeal (D, E, F) mock communities. Panels A and D indicate the effect of sequencing depth on D_MH_ (red bars: large libraries, green bars: small libraries), Panels B/E and C/F indicate the influence of removal of spurious OTUs on D_MH_ for the large libraries (red bars: large libraries, red-hashed bards: large libraries-spurious OTUs removed) and small libraries (green bars: small libraries, green-hashed bars: small libraries-spurious OTUs removed), respectively. The stars indicate D_MH_ values that were significantly different (*p<0.05*).


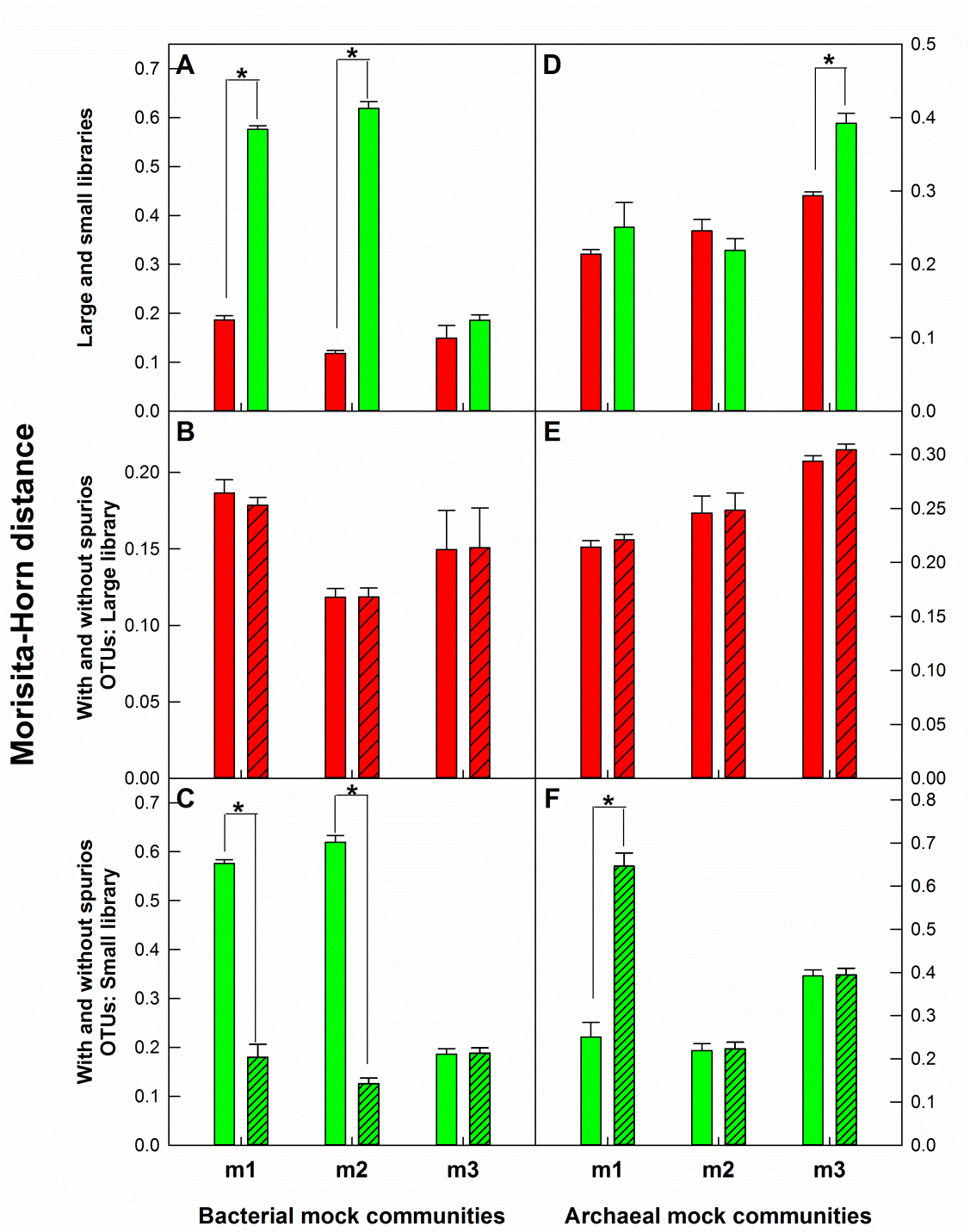

Supplement: Figure S3 — Morisita-Horn distance (DMH) between experimental libraries and theoretical communities for bacterial (A, B, C) and archaeal (D, E, F) mock communities. Panels A and D indicate the effect of sequencing depth on DMH (red bars: large libraries, green bars: small libraries), Panels B/E and C/F indicate the influence of removal of spurious OTUs on DMH for the large libraries (red bars: large libraries, red-hashed bards: large libraries-spurious OTUs removed) and small libraries (green bars: small libraries, green-hashed bars: small libraries-spurious OTUs removed), respectively. The stars indicate DMH values that were significantly different (p<0.05). (DOC) [file pone.0043093.s003.doc]
