# Supplementary material for: PCR Biases Distort Bacterial and Archaeal Community Structure in Pyrosequencing Datasets
Source: PLoS One. 2012 Aug 15;7(8):e43093. doi: 10.1371/journal.pone.0043093 (PMC3419673; doi:10.1371/journal.pone.0043093)

**Figure S4**. Schematic showing workflow from the raw sequencing libraries up to the generation of the “working file” of sequences used for all the results presented in this study. Details for each step are presented in the materials and methods section.


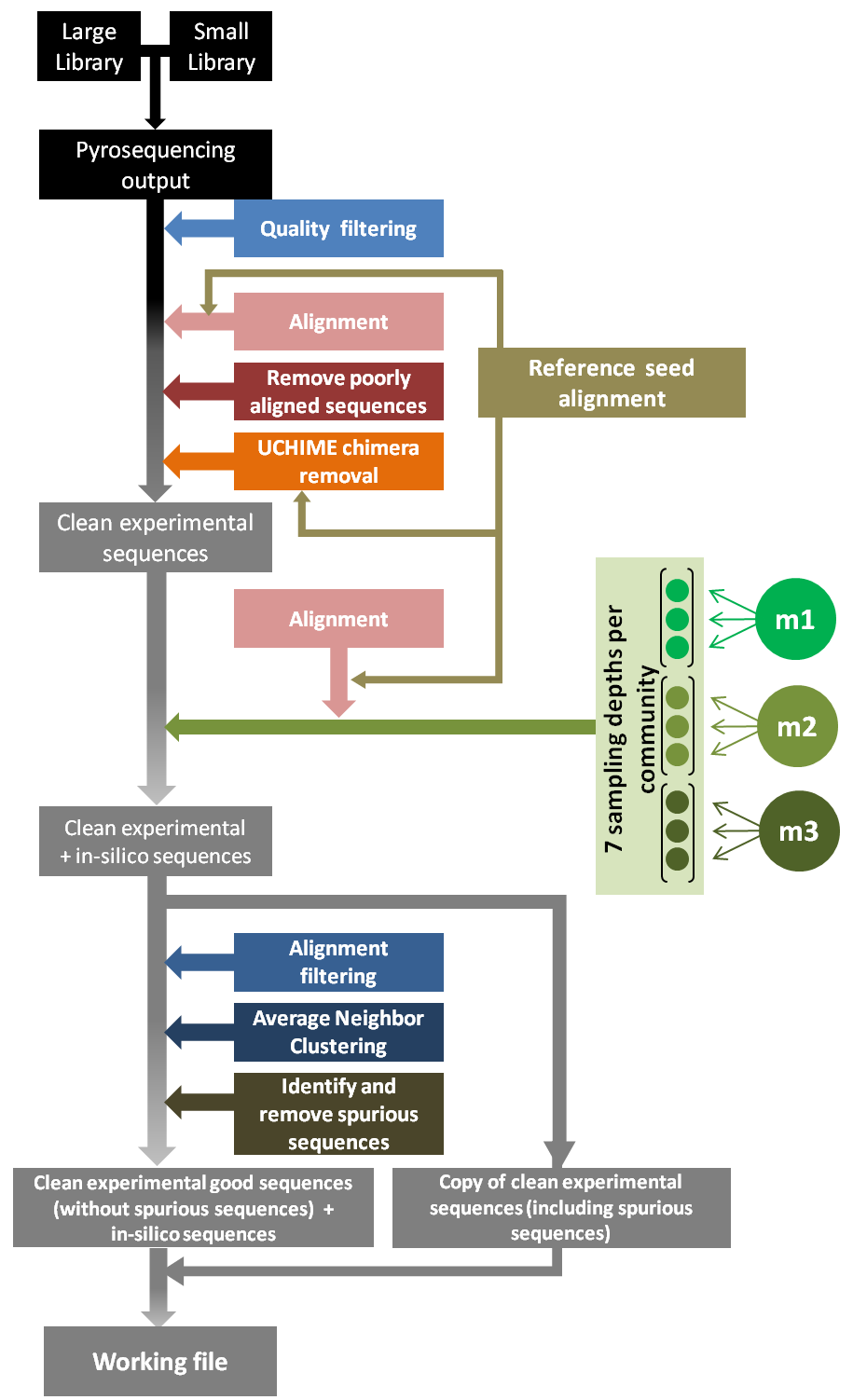

Supplement: Figure S4 — Schematic showing workflow from the raw sequencing libraries up to the generation of the “working file” of sequences used for all the results presented in this study. Details for each step are presented in the materials and methods section. (DOC) [file pone.0043093.s004.doc]
