# Supplementary material for: PCR Biases Distort Bacterial and Archaeal Community Structure in Pyrosequencing Datasets
Source: PLoS One. 2012 Aug 15;7(8):e43093. doi: 10.1371/journal.pone.0043093 (PMC3419673; doi:10.1371/journal.pone.0043093)
Supplement: Table S2 — The detection of bacterial sequences in environmental samples at resolution down to the order level. The classification was conducted on quality filtered and chimera free sequence libraries for each sample. Green boxes indicate sequence classes that were detected. (PDF) [file pone.0043093.s006.pdf]

Table S2: The detection of bacterial sequences in environmental samples at resolution down to the order level. The classification was conducted on quality filtered and chimera free sequence libraries for each sample. Green boxes indicate sequence classes that were detected.

Classification	ANBR	DWDS	MGCT	SW	GC	OP-YNP
**Acidobacteria**	■	■		■	■	
Acidobacteria_Gp21					■	
Acidobacteria_Gp3	■	■		■		
Acidobacteria_Gp4		■				
Acidobacteria_Gp6	■				■	
Acidobacteria_Gp7	■					
Holophagae	■			■		
Holophagales	■			■		
unclassified	■	■				
**Actinobacteria**	■	■	■	■	■	■
Actinobacteria	■	■	■	■	■	■
Acidimicrobidae	■	■		■	■	■
Actinobacteridae	■	■	■	■	■	
Coriobacteridae	■	■	■			
Rubrobacteridae	■	■				
unclassified	■	■		■		
**Aquificae**						■
Aquificae						■
Aquificales						■
**Bacteroidetes**	■	■	■	■	■	
Bacteroidetes_incertae_sedis	■	■		■		
Fulvivirga		■				
unclassified	■					
Bacteroidia	■		■			
Bacteroidales	■		■			
Flavobacteria	■	■		■	■	
Flavobacteriales	■	■		■	■	
Sphingobacteria	■	■		■	■	
Sphingobacteriales	■	■		■	■	
unclassified	■	■		■	■	
unclassified_Bacteroidetes	■	■		■	■	
**Caldiserica**	■					
Caldisericia	■					
Caldisericales	■					
**Chlamydiae**		■				
Chlamydiae		■				
Chlamydiales		■				
**Chloroflexi**	■	■		■	■	
Anaerolineae	■			■	■	
Anaerolineales	■			■	■	
Caldilineae	■					

		Caldilineales						
	Chloroflexi							
		Chloroflexales						
	unclassified							
	unclassified_Chloroflexi							
**Chrysiogenetes**								
	Chrysiogenetes							
		Chrysiogenales						
**Cyanobacteria**								
	Cyanobacteria							
		Chloroplast						
		Family_II						
		Family_XI						
**Deinococcus-Thermus**								
	Deinococci							
		Thermales						
**Firmicutes**								
	Bacilli							
		Bacillales						
		Lactobacillales						
	Clostridia							
		Clostridiales						
		Thermoanaerobacterales						
		unclassified_Clostridia						
	Erysipelotrichi							
		Erysipelotrichales						
	unclassified							
	unclassified_Firmicutes							
**Fusobacteria**								
	Fusobacteria							
		Fusobacteriales						
**Gemmatimonadetes**								
	Gemmatimonadetes							
		Gemmatimonadales						
**Lentisphaerae**								
	Lentisphaeria							
		Victivallales						
		unclassified_Lentisphaeria						
**Nitrospira**								
	Nitrospira							
		Nitrospirales						
**OD1**								
	OD1_genera_incertae_sedis							
**OP10**								
	OP10_genera_incertae_sedis							
**Planctomycetes**								

	Planctomycetacia							
		Planctomycetales						
**Proteobacteria**								
	Alphaproteobacteria							
		Alphaproteobacteria_incertae_sedis						
		Rhizobiales						
		Rhodobacterales						
		Rhodospirillales						
		Rickettsiales						
		Sphingomonadales						
		unclassified						
		unclassified_Alphaproteobacteria						
	Betaproteobacteria							
		Burkholderiales						
		Hydrogenophilales						
		Methylophilales						
		Neisseriales						
		Nitrosomonadales						
		unclassified						
		unclassified_Betaproteobacteria						
	Deltaproteobacteria							
		Bdellovibrionales						
		Desulfobacterales						
		Desulfovibrionales						
		Desulfurellales						
		Desulfuromonadales						
		Myxococcales						
		Syntrophobacterales						
		Syntrophorhabdaceae						
		unclassified						
		unclassified_Deltaproteobacteria						
	Epsilonproteobacteria							
		Campylobacterales						
		unclassified						
	Gammaproteobacteria							
		Alteromonadales						
		Chromatiales						
		Enterobacteriales						
		Gammaproteobacteria_incertae_sedis						
		Legionellales						
		Methylococcales						
		Oceanospirillales						
		Pseudomonadales						
		Thiotrichales						
		Xanthomonadales						
		unclassified						
		unclassified_Gammaproteobacteria						

unclassified						
unclassified_Proteobacteria						
**Spirochaetes**						
Spirochaetes						
Spirochaetales						
Synergistales						
Acholeplasmatales						
unclassified_Mollicutes						
**Verrucomicrobia**						
Opitutae						
Opitutales						
Puniceicoccales						
unclassified						
unclassified_Opitutae						
Spartobacteria						
Spartobacteria_genera_incertae_sedis						
Subdivision3						
Subdivision3_genera_incertae_sedis						
Subdivision5						
Subdivision5_genera_incertae_sedis						
Verrucomicrobiae						
Verrucomicrobiales						
unclassified						
unclassified_Verrucomicrobia						
**WS3**						
WS3_genera_incertae_sedis						
**unclassified**						
**unclassified_Bacteria**						
