# Supplementary material for: PCR Biases Distort Bacterial and Archaeal Community Structure in Pyrosequencing Datasets
Source: PLoS One. 2012 Aug 15;7(8):e43093. doi: 10.1371/journal.pone.0043093 (PMC3419673; doi:10.1371/journal.pone.0043093)
Supplement: Table S3 — The detection of archaeal sequences in environmental samples at resolution down to the family level. The classification was conducted on quality filtered and chimera free sequence libraries for each sample. Green boxes indicate sequence classes that were detected. (PDF) [file pone.0043093.s007.pdf]

Table S3: The detection of archaeal sequences in environmental samples at resolution down to the family level. The classification was conducted on quality filtered and chimera free sequence libraries for each sample. Green boxes indicate sequence classes that were detected.

Classification	ANBR	FAS	GC	OP-YNP
Crenarchaeota	■	■	■	■
Thermoprotei	■		■	■
Caldisphaerales				■
Caldisphaeraceae				■
Desulfurococcales			■	■
Desulfurococcaceae				■
Sulfolobales			■	■
Sulfolobaceae			■	■
Thermoproteales				■
Thermoproteaceae				■
unclassified	■		■	■
env_sample		■		
Euryarchaeota	■		■	■
Archaeoglobi				■
Archaeoglobales				■
Archaeoglobaceae				■
Marine_Group_II			■	
env_sample			■	
Marine_Group_III			■	
env_sample			■	
Methanobacteria	■			
Methanobacteriales	■			
Methanobacteriaceae	■			
Methanomicrobia	■			
Methanomicrobiales	■			
Genera_incertae_sedis	■			
Methanospirillaceae	■			
Methanosarcinales	■			
Methanosaetaceae	■			
Methanosarcinaceae	■			
Thermoplasmata				■
Thermoplasmatales				■
Picrophilaceae				■
Nanoarchaeota				■
env_sample				■
Thaumarchaeota			■	
marine_archaeal_group_1			■	
Nitrosopumilales			■	
Nitrosopumilaceae			■	
env_sample			■	

env_sample				
unclassified				
