# Supplementary material for: PCR Biases Distort Bacterial and Archaeal Community Structure in Pyrosequencing Datasets
Source: PLoS One. 2012 Aug 15;7(8):e43093. doi: 10.1371/journal.pone.0043093 (PMC3419673; doi:10.1371/journal.pone.0043093)
Supplement: Table S4 — Information about the source of each sequence, the length of near full-length 16S rRNA gene sequences amplified, the length of the V3–V5 region, and the GC content and length of the longest homopolymer in the full length and V3–V5 region. (DOC) [file pone.0043093.s008.doc]

**Table S4:** Information about the source of each sequence, the length of near full length 16S rRNA gene sequences amplified, the length of the V3-V5 region, and the GC content and length of the longest homopolymer in the full length and V3-V5 region.

| **Sequence name** | **Source** | **Insert length** | **Insert GC content (%)** | **Insert MaxHomoP** | **V3-V5 length** | **V3-V5 GC content (%)** | **V3-V5 MaxHomoP** |
| --- | --- | --- | --- | --- | --- | --- | --- |
| **BACTERIAL SEQUENCES** | | | | | | | |
| *Cyanobacteria* | *Surface water* | 1324 | 48 | 6 | 569 | 47 | 6 |
| *Mycoplasma orale* | *DSMZ culture collection-1915* | 1375 | 49 | 5 | 579 | 49 | 4 |
| *Bacteroidetes clone 1* | *Wastewater reactor* | 1355 | 51 | 5 | 580 | 50 | 4 |
| *Protochlamydia amoebophilia* | *Donated by Mathias Horn, University of Vienna* | 1360 | 51 | 8 | 590 | 51 | 5 |
| *Chlorobi* | *Surface water* | 1374 | 52 | 5 | 577 | 52 | 5 |
| *Victivallis vadensis* | *DSMZ culture collection-8748* | 1360 | 53 | 6 | 565 | 52 | 5 |
| *Acidobacteria* | *Drinking water* | 1359 | 52 | 5 | 566 | 53 | 4 |
| *Desulfurispirillum alkaliphilum* | *DSMZ culture collection-1827* | 1375 | 55 | 6 | 590 | 53 | 6 |
| *Syntrophococcus sucromutans* | *Donated by Syed Hashsham, Michigan State University (ATCC# 43584)* | 1380 | 55 | 6 | 564 | 53 | 5 |
| *Leptotrichia hofstadii* | *DSMZ culture collection-21561* | 1367 | 55 | 5 | 565 | 53 | 5 |
| *Actinobacteria* | *Wastewater reactor* | 1392 | 52 | 5 | 582 | 49 | 4 |
| *Syntrophus gentianae* | *Donated by Syed Hashsham, Michigan State University (DMZ# 8423)* | 1412 | 53 | 6 | 591 | 53 | 6 |
| *Syntrophomonas bryantii* | *Donated by Syed Hashsham, Michigan State University (DSMZ# 314A)* | 1412 | 55 | 6 | 586 | 54 | 5 |
| *Syntrophus buswellii* | *Donated by Syed Hashsham, Michigan State University (DSMZ# 2612A)* | 1413 | 54 | 6 | 591 | 54 | 6 |
| *Bacteroidetes clone 2* | *Drinking water* | 1352 | 54 | 5 | 566 | 55 | 4 |
| *Caldisericum exile* | *DSMZ culture collection-13637* | 1426 | 55 | 6 | 589 | 55 | 6 |
| *Deinococcus indicus* | *DSMZ culture collection-1537* | 1366 | 56 | 5 | 574 | 55 | 4 |
| *Planctomycetes* | *Wastewater reactor* | 1376 | 56 | 6 | 587 | 55 | 5 |
| *Spirochaetes* | *Surface water* | 1396 | 55 | 5 | 586 | 55 | 5 |
| *Synergistetes* | *Surface water* | 1355 | 55 | 5 | 565 | 55 | 5 |
| *Verrucomicrobia* | *Surface water* | 1379 | 55 | 6 | 590 | 55 | 6 |
| *Persephonella hydrogeniphiia H3* | *Donated by Anne Louise Reysenbach, Portland State University* | 1389 | 60 | 6 | 564 | 60 | 6 |
| *Fibrobacter succinogenes S85* | *Donated by Isaac Cann, University of Illinois-Urbana Champaign* | 1372 | 58 | 5 | 579 | 56 | 5 |
| *Syntrophothermus lipocalidus* | *Donated by Syed Hashsham, Michigan State University (DSMZ# 1268)* | 1500 | 57 | 6 | 590 | 57 | 6 |
| *Syntrophobacter fumaroxidans* | *Donated by Syed Hashsham, Michigan State University (DSMZ# 117)* | 1415 | 57 | 5 | 591 | 57 | 5 |
| *Nitrospira* | *Wastewater reactor* | 1376 | 57 | 6 | 576 | 57 | 5 |
| *Deferribacter desulfuricans* | *DSMZ culture collection-14783* | 1410 | 59 | 5 | 589 | 59 | 5 |
| *Dictyoglomus thermophilum* | *DSMZ culture collection-396* | 1415 | 59 | 5 | 563 | 59 | 5 |
| *Sulfurihydrogenibium yellowstonense* | *Donated by Anne Louise Reysenbach, Portland State University* | 1378 | 57 | 6 | 564 | 56 | 6 |
| *Gemmatimonadetes* | *Wastewater reactor* | 1360 | 60 | 5 | 582 | 61 | 5 |
| *Thermodesulfobacterium commune* | *DSMZ culture collection-2178* | 1422 | 61 | 6 | 588 | 61 | 6 |
| *Thermotoga neapolitana* | *Donated by Claire Vielle, Michigan State University* | 1412 | 64 | 6 | 587 | 62 | 6 |
| *Thermomicrobium roseum* | *DSMZ culture collection-5159* | 1371 | 66 | 6 | 567 | 67 | 6 |
| **ARCHAEAL SEQUENCES** | | | | | | | |
| *Ferroplasma acidiphilum* | *DSMZ culture collection-12658* | 1261 | 51 | 5 | 556 | 51 | 5 |
| *Ferroplasma* | *Yellowstone National Park, DNA extract donated by Mircea Podar, Oak Ridge National Laboratories* | 1344 | 53 | 5 | 556 | 51 | 5 |
| *Methanosphaera stadtmanae* | *Donated by Syed Hashsham, Michigan State University (DSMZ# 3091)* | 1267 | 52 | 6 | 558 | 52 | 6 |
| *Nitrosopumilus maritimus* | *Donated by David Stahl, University of Washington* | 1346 | 53 | 5 | 557 | 52 | 5 |
| *Methanobrevibacter smithii* | *Donated by Syed Hashsham, Michigan State University (DSMZ# 861)* | 1268 | 54 | 6 | 558 | 54 | 6 |
| *Thermoplasma* | *Yellowstone National Park, DNA extract donated by Mircea Podar, Oak Ridge National Laboratories* | 1346 | 55 | 7 | 557 | 54 | 7 |
| *Methanobrevibacter arboriphilus* | *Donated by Syed Hashsham, Michigan State University (DSMZ# 1536)* | 1270 | 54 | 8 | 558 | 55 | 6 |
| *Methanobacterium bryantii* | *Donated by Syed Hashsham, Michigan State University (DSMZ# 862)* | 1266 | 56 | 7 | 558 | 56 | 7 |
| *Methanococcus maripaludis* | *Donated by Syed Hashsham, Michigan State University (DSMZ# 267)* | 1262 | 57 | 6 | 556 | 58 | 6 |
| *Methanolacinia paynteri* | *Donated by Syed Hashsham, Michigan State University (DSMZ# 2545)* | 1261 | 56 | 5 | 554 | 58 | 5 |
| *Methanohalophilus halophilus* | *Donated by Syed Hashsham, Michigan State University (DSMZ# 394)* | 1265 | 56 | 5 | 558 | 58 | 5 |
| *Methanosarcina barkeri* | *Donated by Syed Hashsham, Michigan State University (DSMZ# 800)* | 1268 | 56 | 5 | 558 | 58 | 5 |
| *Methanosarcina mazei* | *Donated by Syed Hashsham, Michigan State University (DSMZ# 3647)* | 1268 | 57 | 5 | 558 | 58 | 5 |
| *Methanosarcina thermophila* | *Donated by Syed Hashsham, Michigan State University (DSMZ# 1825)* | 1268 | 57 | 5 | 558 | 58 | 5 |
| *Methanospirillum hungatei* | *Donated by Syed Hashsham, Michigan State University (DSMZ# 864)* | 1271 | 56 | 5 | 564 | 59 | 5 |
| *Methanothermobacter thermoautotrophicus* | *Donated by Syed Hashsham, Michigan State University (DSMZ# 1910)* | 1271 | 59 | 5 | 559 | 59 | 5 |
| *Halomicrobium katesii* | *DSMZ culture collection-19301* | 1270 | 59 | 5 | 559 | 67 | 5 |
| *Thermoprotei* | *Yellowstone National Park, DNA extract donated by Mircea Podar, Oak Ridge National Laboratories* | 1362 | 62 | 6 | 556 | 62 | 6 |
| *Metallosphaera* | *Yellowstone National Park, DNA extract donated by Mircea Podar, Oak Ridge National Laboratories* | 1367 | 63 | 6 | 553 | 62 | 6 |
| *Caldivirga maquilingensis* | *DSMZ culture collection-13496* | 1461 | 65 | 7 | 733 | 64 | 7 |
| *Sulfolobus acidocaldarius* | *Donated by David Stahl, University of Washington (DSMZ #: 639)* | 1278 | 63 | 6 | 553 | 65 | 6 |
| *Thermocladium* | *Yellowstone National Park, DNA extract donated by Mircea Podar, Oak Ridge National Laboratories* | 1377 | 63 | 7 | 557 | 65 | 7 |
| *Archaeoglobus profundus* | *DSMZ culture collection-5631* | 1288 | 65 | 6 | 558 | 67 | 5 |
| *Thermococcus guaymasensis* | *DSMZ culture collection-11113* | 1269 | 66 | 6 | 557 | 67 | 6 |
