# Supplementary material for: PCR Biases Distort Bacterial and Archaeal Community Structure in Pyrosequencing Datasets
Source: PLoS One. 2012 Aug 15;7(8):e43093. doi: 10.1371/journal.pone.0043093 (PMC3419673; doi:10.1371/journal.pone.0043093)
Supplement: Table S5 — The number of sequences obtained for each bacterial and archaeal mock community before and after quality filtering and chimera removal. Sm- small library generated during first sequencing run, lg-large library generated during second sequencing run. (DOC) [file pone.0043093.s009.doc]

**Table S5:** The number of sequences obtained for each bacterial and archaeal mock community before and after quality filtering and chimera removal. Sm- small library generated during first sequencing run, lg-large library generated during second sequencing run.

|  | **Sample** | **Original sequences** | **Quality filtered, chimera free sequences** | **Sequences removed (%)** |
| --- | --- | --- | --- | --- |
|  | **BACTERIA** | | | |
| **First run** | m1.1-sm | 524 | 446 | 14.9 |
|  | m1.2-sm | 636 | 566 | 11.0 |
|  | m1.3-sm | 401 | 356 | 11.2 |
|  | m2.1-sm | 618 | 570 | 7.8 |
|  | m2.2-sm | 572 | 543 | 5.1 |
|  | m2.3-sm | 580 | 546 | 5.9 |
|  | m3.1-sm | 396 | 354 | 10.6 |
|  | m3.2-sm | 545 | 489 | 10.3 |
|  | m3.3-sm | 508 | 449 | 11.6 |
| **Second run** | m1.1-lg | 2631 | 2268 | 13.8 |
|  | m1.2-lg | 3410 | 3011 | 11.7 |
|  | m1.3-lg | 2986 | 2653 | 11.2 |
|  | m2.1-lg | 3833 | 3604 | 6.0 |
|  | m2.2-lg | 3636 | 3426 | 5.8 |
|  | m2.3-lg | 3783 | 3562 | 5.8 |
|  | m3.1-lg | 2635 | 2340 | 11.2 |
|  | m3.2-lg | 3936 | 3505 | 11.0 |
|  | m3.3-lg | 3686 | 3269 | 11.3 |
| **Sequences removed by quality filtering and chimera checking (%)** | | | | 9.8±2.9 |

|  | **Sample** | **Original sequences** | **Quality filtered, chimera free sequences** | **Sequences removed (%)** |
| --- | --- | --- | --- | --- |
|  | **Archaea** | | | |
| **First run** | m1.1-sm | 326 | 284 | 12.9 |
|  | m1.2-sm | 308 | 286 | 7.1 |
|  | m1.3-sm | 285 | 248 | 13.0 |
|  | m2.1-sm | 427 | 378 | 11.5 |
|  | m2.2-sm | 503 | 447 | 11.1 |
|  | m2.3-sm | 391 | 357 | 8.7 |
|  | m3.1-sm | 165 | 152 | 7.9 |
|  | m3.2-sm | 224 | 206 | 8.0 |
|  | m3.3-sm | 171 | 151 | 11.7 |
| **Second run** | m1.1-lg | 1901 | 1578 | 17.0 |
|  | m1.2-lg | 1563 | 1349 | 13.7 |
|  | m1.3-lg | 1746 | 1479 | 15.3 |
|  | m2.1-lg | 2113 | 1792 | 15.2 |
|  | m2.2-lg | 3412 | 2957 | 13.3 |
|  | m2.3-lg | 2386 | 2012 | 15.7 |
|  | m3.1-lg | 1094 | 960 | 12.2 |
|  | m3.2-lg | 1417 | 1266 | 10.7 |
|  | m3.3-lg | 1232 | 1114 | 9.6 |
| **Sequences removed by quality filtering and chimera checking (%)** | | | | 11.9±2.9 |
