# Supplementary material for: PCR Biases Distort Bacterial and Archaeal Community Structure in Pyrosequencing Datasets
Source: PLoS One. 2012 Aug 15;7(8):e43093. doi: 10.1371/journal.pone.0043093 (PMC3419673; doi:10.1371/journal.pone.0043093)
Supplement: Table S6 — Primers used to amplify near full-length 16S rRNA gene sequences used to generate the mock communities. (DOC) [file pone.0043093.s010.doc]

**Table S6**: Primers used to amplify near full length 16S rRNA gene sequences used to generate the mock communities.

| **Primer Name** | **Primer Sequence (5'-3')** | **Target region**  **(E.Coli numbering)** | **Primer used for** | **Reference** |
| --- | --- | --- | --- | --- |
| Bact-8F modified | AGRGTTTGATCMTGGCTCAG | 8-27 | All bacteria-except *Protochlamydia amoebophila* | This study |
| Bact-1387R modified | GGGCGGWGTGTACAAGRC | 1369-1387 | All bacterial sequences | This study |
| Chlam-Forward | CGTGGATGAGGCATGCAAGTCGA | 44-56 | *Protochlamydia amoebophilia* | This study |
| Arch-4F | TCCGGTTGATCCTGCCRG | 4-21 | Yellowstone clones | 1 |
| Arc-112F modified | GCTSAGTAACACGTSG | 112-128 | All archaeal pure cultures | This study |
| Univ-1406R modified | GACGGGCGGTGWGTRCA | 1406-1424 | All archaeal sequences | This study |
| Kora 228F | GAGGCCCCAGGRTGGGACCG | 228-248 | Check korarchaeal presence in Yellowstone sample | 2 |
| Kora 1236R | CATCCCGCTGTCCCGCCCATTGC | 1200-1223 | Check korarchaeal presence in Yellowstone sample | 3 |
| Bact338f old | CCTACGGGAGGCAGCAG | 341-357 | Previously published forward primer for bacterial 16S rRNA gene | 4 |
| Bact909r old | CCGTCAATTCMTTTRAGT | 909-926 | Previously published forward primer for bacterial 16S rRNA gene | 4 |
| Arch340F | CCCTACGGGGYGCASCAG | 340-358 | Previously published forward primer for archaeal 16S rRNA gene | 2, 5 |
| Arch934R | GTGCTCCCCCGCCAATTCCT | 914-934 | Previously published forward primer for archaeal 16S rRNA gene | 2, 5 |

1. Reysenbach A, Ehringer M, Hershberger K (2000) Microbial diversity at 83°C in Calcite Springs, Yellowstone National Park: another environment where the “Aquificales” and "Korarchaeota" coexist*.* Extremophiles 4: 61-67.

2. Baker G, Smith J, Cowan D (2003) Review and re-analysis of domain-specific 16S primers. J Microbiol Methods 55: 541-555.

3. Auchtung TA, Takacs-Vesbach CD, Cavanaugh CM (2006) 16S rRNA phylogenetic investigation of the candidate division "*Korarchaeota*". Appl Environ Microbiol 72: 5077-5082.

4. Haas B, Gevers D, Earl A, Feldgarden M, Ward D, et al. (2011) Chimeric 16S rRNA sequence formation and detection in Sanger and 454-pyrosequenced PCR amplicons. Genome Res 21: 494-504.

5. Stahl DA, Amann R (1991) Development and application of nucleic acid probes. In: Stackebrandt E, Goodfellow M, editors. Nucleic acid techniques in bacterial systematics. Chichester, England: John Wiley & Sons. pp. 205-248.
